# Single-Ion Anisotropy and Intramolecular Interactions in Ce^III^ and Nd^III^ Dimers

**DOI:** 10.1021/acs.inorgchem.1c00647

**Published:** 2021-06-10

**Authors:** Júlia Mayans, Lorenzo Tesi, Matteo Briganti, Marie-Emmanuelle Boulon, Mercè Font-Bardia, Albert Escuer, Lorenzo Sorace

**Affiliations:** † Departament de Química Inorgànica i Orgànica, Secció Inorgànica and Institute of Nanoscience and Nanotechnology (IN^2^UB), Universitat de Barcelona, Martí i Franques 1-11, Barcelona-08028, Spain; ‡ Dipartimento di Chimica “Ugo Schiff” & INSTM RU, Università degli Studi di Firenze, Via della Lastruccia 3-13, 50019 Sesto Fiorentino (Firenze), Italy; § Unitat de Difracció de R-X, Centre Científic i Tecnològic de la Universitat de Barcelona (CCiTUB), Universitat de Barcelona, Solé i Sabarís 1-3, 08028 Barcelona, Spain

## Abstract

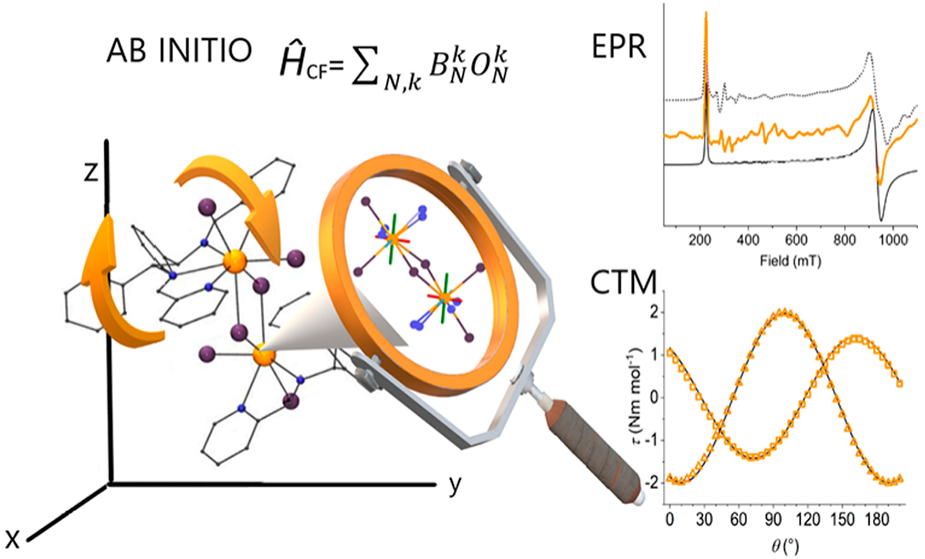

This article reports the syntheses, characterization, structural description, together with magnetic and spectroscopic properties of two isostructural molecular magnets based on the chiral ligand *N*,*N*′-*bis*((1,2-diphenyl-(pyridine-2-yl)methylene)-(*R*,*R*/*S*,*S*)-ethane-1,2-diamine), **L1**, of general formula [Ln_2_(*RR*-**L1**)_2_(Cl_6_)]**·**MeOH**·**1.5H_2_O, (Ln = Ce (**1**) or Nd (**2**)). Multifrequency electron paramagnetic resonance (EPR), cantilever torque magnetometry (CTM) measurements, and ab initio calculations allowed us to determine single-ion magnetic anisotropy and intramolecular magnetic interactions in both compounds, evidencing a more important role of the anisotropic exchange for the Nd^III^ derivative. The comparison of experimental and theoretical data indicates that, in the case of largely rhombic lanthanide ions, ab initio calculations can fail in determining the orientation of the weakest components, while being reliable in determining their principal values. However, they remain of paramount importance to set the analysis of EPR and CTM on sound basis, thus obtaining a very precise picture of the magnetic interactions in these systems. Finally, the electronic structure of the two complexes, as obtained by this approach, is consistent with the absence of zero-field slow relaxation observed in ac susceptibility.

## Abstract 2

Multifrequency electron paramagnetic resonance (EPR), cantilever torque magnetometry (CTM) measurements, and ab initio calculations lead to determination of both the single-ion magnetic anisotropy and the intramolecular magnetic interactions in two new chloro-bridged Nd^III^ and Ce^III^ dimers. The combination of these techniques appears to be fundamental for determining the weak intramolecular exchange interactions characterizing lanthanide-based polynuclear complexes, which play a subtle but relevant role in determining the magnetic properties of these systems.

## Introduction

The discovery by Ishikawa and co-workers^1^ of slow magnetic relaxation in terbium-phthalocyaninate provoked a growing interest in the use of lanthanide complexes as single molecule magnets (SMMs)^2−6^ and, more recently, for quantum information processing (QIP).^7−10^ The interesting magnetic behavior of these complexes benefits from their large unquenched orbital angular moments, resulting from strong spin–orbit coupling and weak ligand fields, which impart strong magnetic anisotropy.

Looking forward to these ambitious applications, it is important to understand all the possible variables that can play a relevant role in determining the dynamic magnetic properties of these molecular systems. In this framework, the intramolecular exchange interaction between lanthanide (Ln) ions is a still scarcely investigated parameter,^11−16^ because of the complexity of its determination.^17^ The exchange interaction strongly influences the magnetization relaxation pathways of SMMs, e.g., by reducing the effect of quantum tunneling of magnetization (QTM) at zero-field. This effect is analogous to exchange bias in permanent magnets, thus allowing zero-field magnetic remanence.^18^ On the other hand, the observed relaxation under an applied field is often faster in weakly exchange-coupled systems than in mononuclear ones, as a consequence of state mixing induced by the interaction.^19^ The use of this exchange-bias approach to reduce the effectiveness of tunneling is applicable for weak 4f–4f interactions. In contrast, stronger interactions yield a coupled system behaving as a single giant spin.^20,21^ This requires the presence of additional 2p or 3d paramagnetic centers,^22,23^ resulting in a slowdown of the relaxation. Indeed, a multistep Orbach process is favored, while competitive processes such as Raman and direct ones become less efficient.^20,24^ An intramolecular exchange interaction between two Ln ions can also be a way to achieve entanglement between two spins, which is required for the realization of two-quantum-bit gates.^25,26^ This approach presents the advantage of being spatially precise as the distance between the two spins is imposed by the molecular structure. However, this interaction must be finely tuned, with respect to the anisotropic features of the constitutive ions, and this, in turn, requires a deep understanding of the factors affecting it. Obviously, the surroundings of the qubits must also be chosen with care, since the presence of any other spin provides a pathway that accelerates the relaxation, limiting their usefulness.

All aforementioned applications require a quantitative interpretation of the magnetic data, in terms of exchange interaction for Ln^III^-based complexes. However, this is not straightforward, since ligand field strength is usually one order of magnitude larger than exchange coupling, and a large deviation from Curie-type behavior is observed, even in the absence of exchange interactions. Furthermore, the isotropic exchange Hamiltonian, which provides a simple conceptual framework to analyze the data in case of orbitally nondegenerate systems, cannot be applied to these systems. Indeed, the orbital degeneracy requires the effective exchange Hamiltonian to be expressed in terms of both spin and orbital operators.^27,28^ Finally, one must consider that magnetic dipolar interactions are of comparable magnitude to superexchange ones, because of the large magnetic moment of Ln^III^ ions. Thus, the exchange interaction in polynuclear Ln^III^ systems cannot be obtained by simple powder magnetic measurements but requires a multitechnique approach that involves magnetic and spectroscopic measurements backed up by ab initio calculations, and possibly the use of single-crystal measurements.^12,13,15^

In this work, we investigate and compare the magnetic properties of two isostructural chiral bimetallic molecular magnets with general formula [Ln_2_(*RR-*L1)_2_(Cl_6_)]**·**MeOH**·**1.5H_2_O, where the [Ln^III^_2_] core is based on Ln = Ce (**1**) or Nd (**2**), and **L1** = *N*,*N*′-*bis*((1,2-diphenyl-(pyridine-2-yl)methylene)-(*R*,*R*/*S*,*S*)-ethane-1,2-diamine).

The choice of *bis*-bidentate Schiff bases ligands originates from the ease of the synthetic procedure, which occurs by condensation of a diamine with two aldehydes or ketones, and from their high versatility. The use of substituted diamines with chiral centers as the starting reagent is a simple and elegant synthetic route for obtaining chiral ligands that can be useful for enantioselective catalysis^29,30^ or for introducing chiroptical or magnetochiral properties in new complexes.^31−35^ On the other hand, the focus on complexes of two early Ln ions stems from the importance of characterizing the exchange interaction for these more-abundant and less-expensive rare-earth ions. Indeed, exchange and anisotropy effects in these elements are at the heart of the remarkable magnetic features of classical hard magnets,^36^ and they offer a great case study for our aim.

To provide a clear picture of the magnetic anisotropy of these systems and to quantify the magnetic exchange interactions, we use a multitechnique investigation including dc magnetometry, continuous wave electron paramagnetic resonance (cw-EPR), and cantilever torque magnetometry (CTM) experiments, flanked by complete active space self consistent field (CASSCF) calculations. This study allows us to discuss, in depth, a methodology to determine the intramolecular interaction and anisotropy with precision. Our results provide a good understanding of complexes of interacting early Ln ions, which is a step forward for improved control and design of such systems.

## Results

### Synthesis

**Scheme 1:**
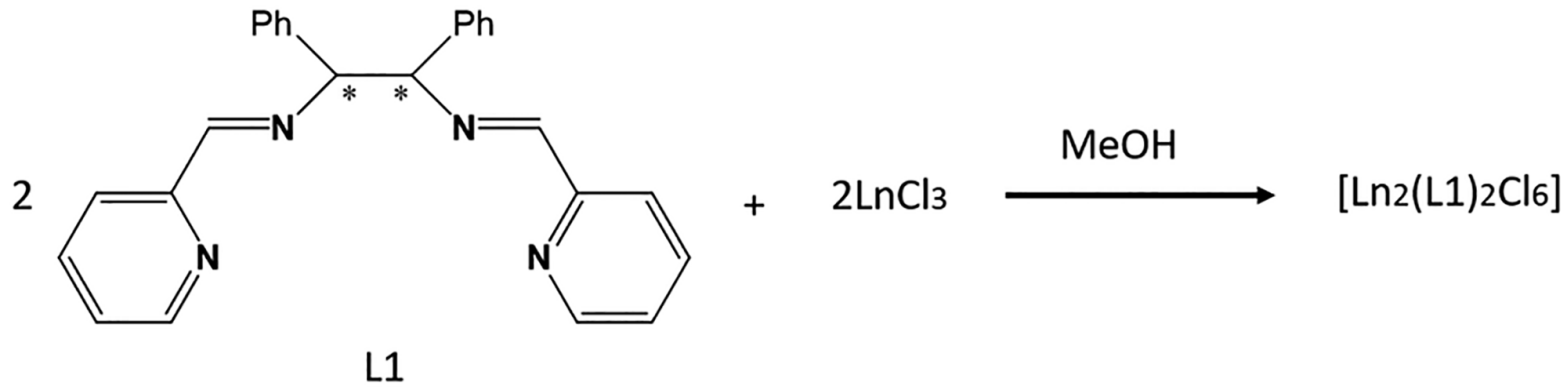


Complexes **1** and **2** were synthesized by direct reaction of the Schiff base with the corresponding lanthanide trichloride in methanolic solution, according to Scheme 1.

The size of the cation determines that dimeric complexes with double chloro bridge and octacoordinated LnN_4_Cl_4_ environment can be obtained for the larger lanthanides (from Ce^III^ to Sm^III^), whereas monomeric complexes with formula [Ln(**L1**)Cl_3_] and LnN_4_Cl_3_ heptacoordination were previously reported for the smaller ones (from Eu^III^ to Yb^III^).^37^ We also tried to obtain the isostructural diamagnetic La^III^ derivative, following the same procedure but all our attempts were unsuccessful. Electronic circular dichroism spectra of the representative pair of dimeric complexes **2***RR*, and **2***SS* show absorptions with positive Cotton effect at λ_max_ = 270 and 221 nm and negative Cotton effect at 300 and 210 nm for **2***RR* and a mirror image for **2***SS*, confirming its enantiomeric identity (see Figure S1 in the Supporting Information).

### Structural Description

Monocrystal X-ray diffraction (XRD) experiments were performed for complexes **1***RR***, 2***RR*, and **2***SS***.** These complexes are isostructural (see Table S1 in the Supporting Information), and a common description is provided in order to avoid repetitions.

#### [Ln_2_(**L1**)_2_(Cl_6_)] (**Ln** = Ce, (*RR*-**L1**), **1***RR*·1.5H_2_O; Ln = Nd, (*RR*-**L1**), **2***RR*·0.5MeOH**·**0.75H_**2**_O; Ln = Nd, SS–(**L1**), 2*SS*·2.5H_2_O)

**Figure 1:**
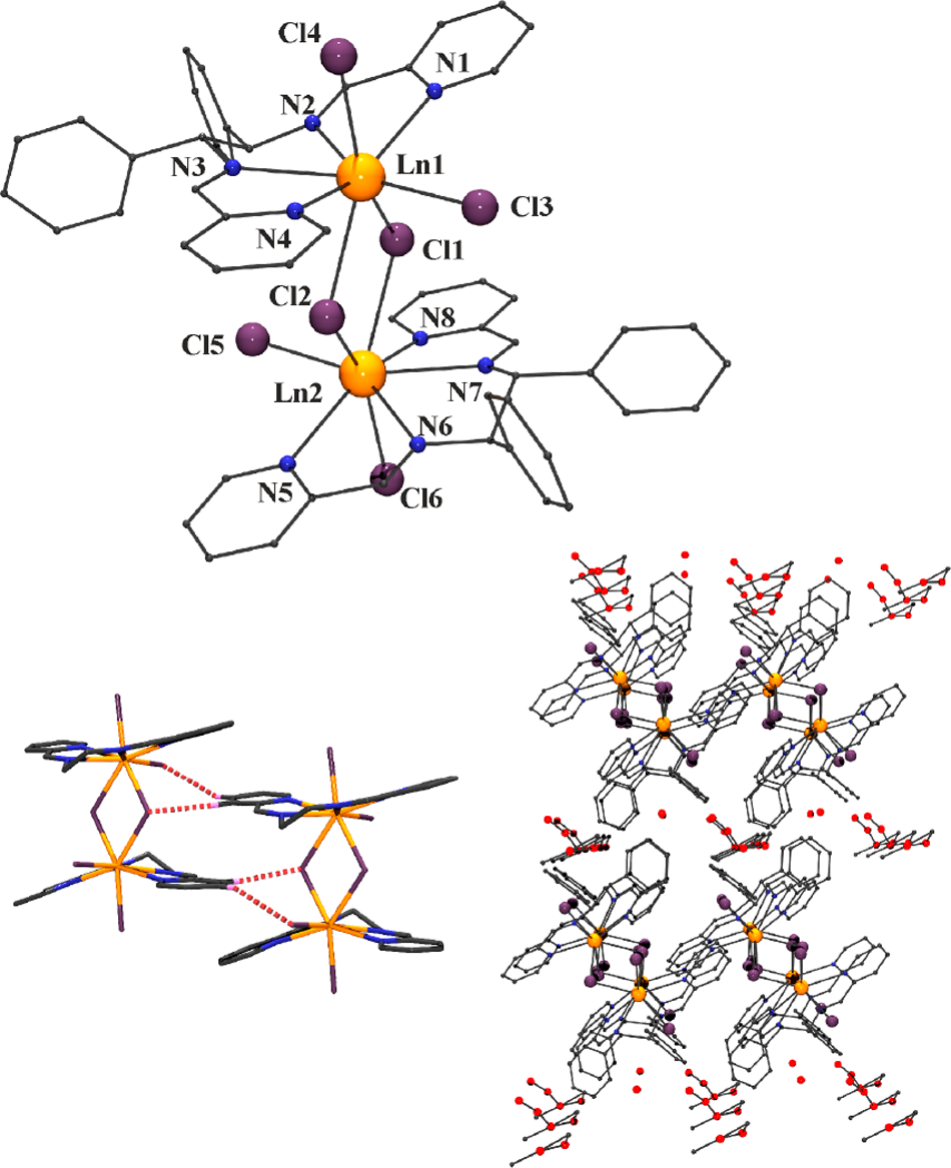
Top: Molecular structure of complexes **1** (Ln = Ce) and **2** (Ln = Nd), with the principal atoms labeled. Small gray balls represent C atoms; H atoms have been omitted for the sake of clarity. Bottom: intermolecular hydrogen bonds that determine the arrangement of the dimers and a view of the layers of solvent and dimers in the network.

A view of the molecular structure is shown in Figure 1 (top), while the main structural parameters are summarized in Table S2 in the Supporting Information.

The structure consists of a neutral Ln^III^ complex in which the two cations are linked by two μ-Cl bridges. Each cation is octacoordinated by the four N atoms of one **L1** ligand, two terminal chloro ligands, and the two bridging chloro donors. The pyridyl groups of **L1** ligand are slightly bent, with respect to the main plane, with the cations placed ∼0.63 Å out of the plane determined by the four N-donors and toward the other cation. The neighbor N-donors of **L1**, linked by one ethylene spacer, determine a narrow bite with N–Ln–N bond angles close to 63°, allowing one of the chloro ligands to be placed in the same plane of the coordination polyhedron. The two μ-Cl bridges and the other terminal chloro ligands are placed in the axial positions above and below the NdN_4_Cl plane. The shorter bond distances correspond to the Nd–N bonds, average ∼2.6 Å, whereas the longer ones are those related to the Ln–(μ)–Cl bonds: these average to 2.85 Å but for each metal center there is clearly a long (2.90 Å) and a short (2.80 Å) Ln–Cl bond. The Ln1–Cl–Ln2 bond angles take a value at ∼109.7°, thus yielding a Ln1–Ln2 intramolecular distance of 4.65 Å. SHAPE^38^ analysis provides evidence that the coordination polyhedra around the two lanthanoid ions are largely distorted, with only a slight preference for bicapped trigonal prisms (BTPR-8, Ln1) and triangular dodecahedra (TDD-8, Ln2), respectively (see Table S3 in the Supporting Information).

According to a survey in the CSD, the LnN_4_Cl_4_ (Ln = Ce^III^, Nd^III^) coordination environment has been previously reported only for a couple of nonpolymeric structures:^39,40^ interestingly, both are dimeric, double chloro-bridged structures. For the noncentrosymmetric structures reported in ref (39) the coordination polyhedra are distorted with slight preference for BTPR-8 and TDD-8, while a square antiprism is clearly favored for the centrosymmetric Nd dimer reported in ref (40). In both cases, bond lengths and angles are consistent with those reported in this study.

A complex set of intramolecular and intermolecular weak CH···Cl and CH···OH interactions relating the dimers and the solvent molecules is observed. Among them, the intramolecular CH···Cl interactions involving H atoms from the pyridinic groups and the bridging chlorides and in-plane terminal chloro ligands are of particular relevance. Indeed, they determine the parallel arrangement of dimers in the network, with layers of dimers separated by layers of crystallization solvents (see bottom of Figure 1).

The powder diffraction spectra of **1** and **2** were performed to check their purity and phase consistence prior to magnetic characterization. The spectra corroborated the isostructurality of the complexes but were surprisingly different from the spectrum expected by the single-crystal data (see Figure S2). To verify if such a difference could be attributed to a loss of solvent, the crystals of the analogous Sm^III^ (**3**) complex, obtained following the same procedure, were maintained at open air for 24 h and single-crystal data were collected after this time.

After 24 h of open-air exposure, the crystals do not visually evidence apparent changes; however, the quality of their diffraction pattern is reduced, thus lowering the quality of the solved molecular structure. Nonetheless, we report it here since it is necessary to interpret the powder diffraction pattern (vide infra) and its main features (unit cell parameters, connectivity, major packing features) can be safely determined. The arrangement of the dimers in 2D planes and bond parameters of the first coordination sphere of the lanthanides are identical in the general trends to the above-described structure for **1** and **2** (Tables S1 and S2). However, the layers of methanol and water molecules of crystallization in the *ab* plane of the nonaged crystals are completely lost (Figure S3 in the Supporting Information): we thus label this complex as **3b**. While its *a*- and *b*-cell parameters are practically identical (9.707(1) and 11.853(1) Å) to those of **1** and **2**, the *c*-parameter of the cell becomes 12.593(1) Å (that is ∼2.6 Å shorter than in the structures containing the layer of solvents). The powder X-ray spectrum (PXRD) simulated from the monocrystal structure of the desolvated form matches perfectly with those obtained from the powdered samples of **1** and **2**, indicating that those are desolvated species (see Figure S2).

### DC Magnetic Characterization

A preliminary investigation of the magnetic properties of **1** and **2** was performed on microcrystalline powder samples. The temperature dependence of the magnetic susceptibility in the 2–300 K range for the two complexes is shown in Figure S4 in the Supporting Information, as χ_M_*T* vs *T* plots. For both molecules, χ_M_*T* at room temperature is close to the free ion value for two corresponding magnetically independent lanthanide cations (^2^F_5/2_ for Ce^III^ and ^4^I_9/2_ for Nd^III^). When the temperature is lowered, the χ_M_*T* product reduces for both compounds, reaching a final value of 0.55 cm^3^ mol^–1^ K for **1** and 1.52 cm^3^ mol^–1^ K for **2**. This decrease can be attributed to the thermal depopulation of the energy levels of the ground multiplet split by Crystal Field effects. At the lowest temperature, intramolecular interactions, which are expected to be rather weak in these systems, might also contribute.

The magnetic-field-dependent magnetization was measured in the range of 0–5 T and at three different temperatures (see Figure S5 in the Supporting Information). Magnetization does not saturate, and the reduced magnetization shows quasi-superimposable plots: this indicates an almost-complete population of the ground doublet.

### Electron Paramagnetic Resonance

EPR is a useful tool for investigating the magnetic anisotropy and exchange interactions of lanthanides with an odd number of unpaired electrons, such as Ce^III^ and Nd^III^.^13,41,42^ For this reason, powder samples of complexes **1** and **2** were investigated at helium temperature, using cw-EPR at two frequencies (∼9 GHz for the X-band and ∼94 GHz for the W-band).

**Figure 2:**
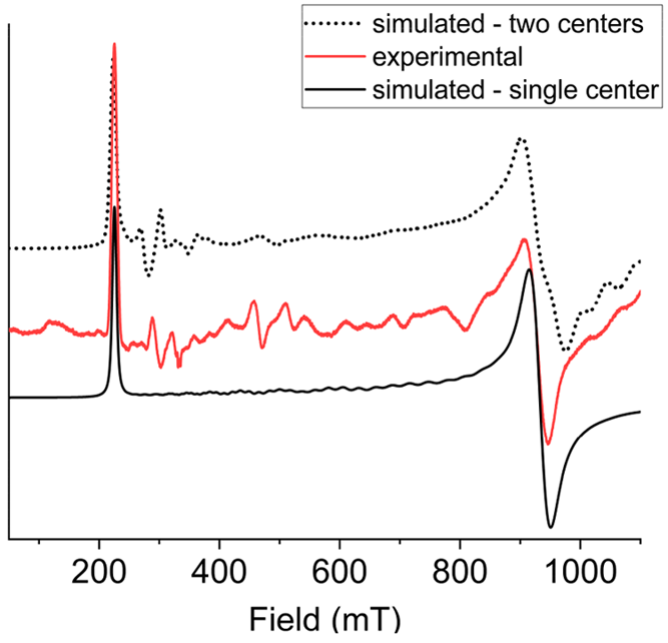
Experimental (red) and simulated (black) X-band EPR spectrum of a polycrystalline powder sample of compound **1** measured at 5 K. Dashed line represents the simulated spectrum by assuming noncollinear single-ion anisotropy and calculated dipolar interactions.

Figure 2 shows the experimental powder EPR spectra of complex **1** at 5 K in the X-band. The spectrum presents a first absorption band at ∼200 mT and a second broader one at ∼950 mT. The shape of the spectrum is as expected for a single, well-isolated anisotropic doublet, where the second line is the component of a rhombic doublet rather than the perpendicular one in an axial system. On the other hand, there are no evidence of intramolecular exchange or dipolar interactions, nor any indication about difference in magnetic behavior between the two structurally inequivalent lanthanide centers.

A simulation of the EPR spectrum of **1** could then be obtained by assuming an effective Spin Hamiltonian,
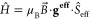
1where μ_B_ is the Bohr magneton,  the effective spin angular moment,  the dc magnetic field, and **g^eff^** the effective Landé **g** tensor of the ground doublet. A good agreement with the experimental data is found assuming as principal values of **g^eff^**, *g*_*x*_ < 0.5, *g*_*y*_ = 0.72, and *g*_*z*_*=* 2.98. Note that *g*_*x*_ is not observed as it occurs at magnetic fields outside our accessible field range.

**Figure 3:**
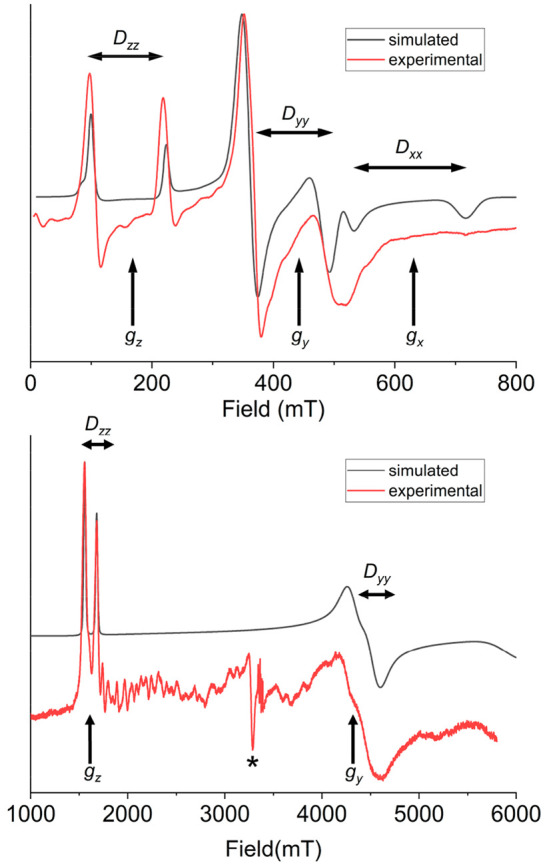
Experimental (red lines) and simulated (black lines) powder EPR spectra of **2**, measured at the X-band and 5 K (top) and at the W-band and 14 K (bottom). Simulations are performed using the Hamiltonian of eq 2 and assuming a collinear single-ion anisotropy. The asterisk marks an unavoidable cavity impurity in the W-band spectrometer.

For the Nd^III^ derivative, complex **2**, the powder X-band EPR spectrum at 5 K is depicted in the top portion of Figure 3. The observed number of spectral lines is clearly not compatible with a single effective ground doublet with **g^eff^** anisotropy. The extra bands in the spectrum can be, in principle, due to the presence of low-lying excited states (which can allow intradoublet transitions), to intramolecular magnetic interactions (either dipolar or exchange in nature), or to appreciable differences in the magnetic features of the two magnetic centers. The three hypotheses can be verified by measuring the spectrum at a different frequency, since each of them would result in a completely different pattern. Indeed, if the splitting between lines is due to intramolecular interactions, it should be frequency-independent. On the other hand, if the separation is due to intermultiplet transitions within each center or to different magnetic anisotropy of the two structurally inequivalent lanthanides, it should vary on varying frequency.

W-band EPR spectrum (ν ∼ 94 GHz) on a powder sample was then performed at 14 K (Figure 3, bottom). The splitting of the two lowest field lines is the same observed at low frequency. This suggests a non-negligible intramolecular interaction between the anisotropic doublets. Following this interpretation, we simulated the spectra at the two frequencies, on the basis of the following spin Hamiltonian:
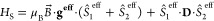
2where we assumed the two Nd^III^ centers as equivalent, with an effective spin *S* = 1/2, and **D** is a traceless interaction tensor collinear to **g^eff^**. In this approach, each pair of lines is centered at *g*_*i*_ (*i* = *x*, *y*, *z*) with a separation of *D*_*ii*_/*g*_*i*_μ_B_. The best simulation was obtained with principal **g^eff^** values of *g*_*z*_ = 4.16 *g*_*y*_ = 1.52 *g*_*x*_ = 1.08 and a traceless anisotropic interaction between Nd^III^ ions with principal values **D** = [0.08 0.08 −0.16] cm^–1^. It is worth stressing that, due to the assumption of collinearity between the two centers, the inclusion of an isotropic exchange coupling in eq 2 does not change the spectral appearance, but only the intensity of the simulated spectrum, as a function of temperature. Because of the low magnitude of the isotropic coupling, these effects are however not large enough to allow its determination.

The traceless nature of the **D** tensor would suggest a dipolar nature of the interaction in **2**. However, its principal values are ca. 50% larger than those calculated on the basis of point dipole approximation and structural parameters, even in the assumption of the maximum **g^eff^** component being oriented along the Nd1–Nd2 direction, which is not demonstrated at this stage. This suggests that further contributions, mainly due to anisotropic exchange, are not negligible. To clarify this point it is necessary to know the orientation of the **g^eff^** tensor with respect to the molecular frame, which is a problem that we tackled using CTM and ab initio calculations.

### Cantilever Torque Magnetometry

CTM allows one to determine the magnetic anisotropy of coordination compounds and its correlation with the molecular structure. It is especially useful when applied to low-symmetry lanthanide complexes, because of the not-easily predictable nature and orientation of their magnetic anisotropy.^43−45^

**Figure 4:**
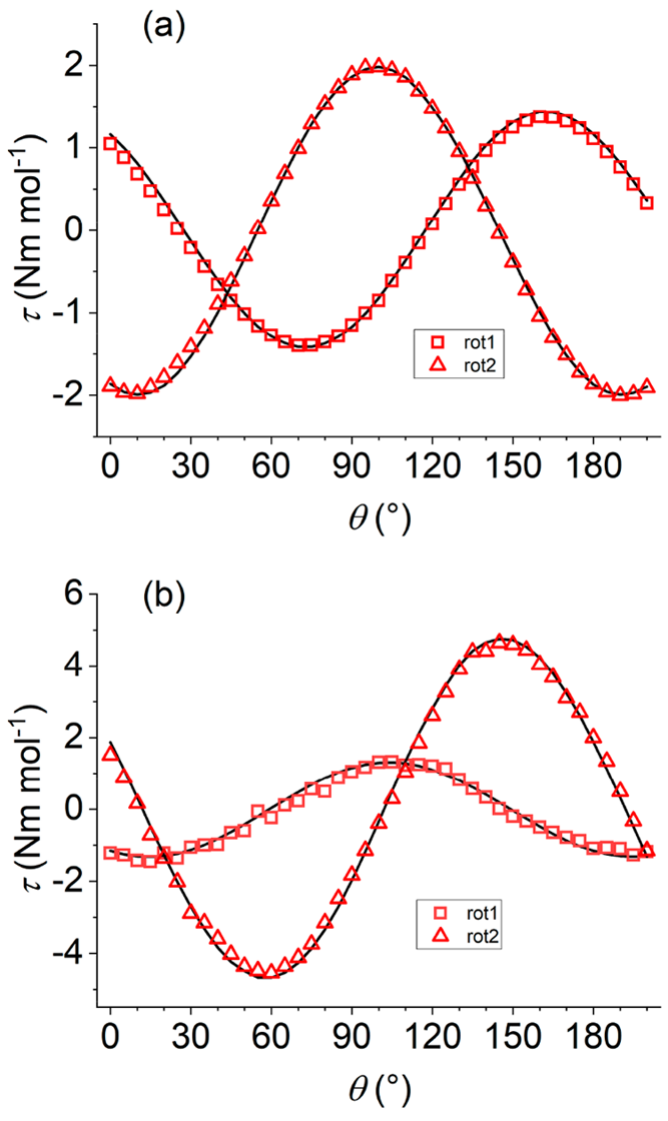
Experimental and best fit CTM data for **1** (upper) and **2** (lower) at 2 K and 1 T using the principal **g^eff^** values from EPR and leaving the orientation of the anisotropy axis free to refine. The best-fit orientations are shown in Figure 5.

With this aim, single crystals of the two compounds were indexed by XRD and placed on the cantilever. To reconstruct the principal magnetic tensors, rotations around two orthogonal axes were performed under a static magnetic field with a fixed orientation (Rot1 and Rot2; see Figure 4) perpendicular to the rotation axis. The initial position of the crystal, with respect to the laboratory frame in each of the two rotations, is described in Figure S6 in the Supporting Information. During the experiment, the crystal is rotated and each time the magnetic field is parallel to the projection of one principal magnetic axis on the scanned plane, then the measured torque component is null: τ_Y_ = *M*_*z*_*B*_*x*_ – *M*_*x*_*B*_*z*_ = 0. For the first rotation, Rot1, this occurs at θ = 27° and 117° for compound **1** and for compound **2** at θ = 60° and 150°; in both cases, the expected 90° periodicity is fulfilled (see Figure 4). The complete set of experimental data and the corresponding fits are reported in Figures S7 and S8 in the Supporting Information.

For the second rotation measurement set, Rot2, the crystals were first manually rotated by 90° on the sample holder with respect to Rot1 configuration and the experiment was repeated: for **1**, the zeros of the torque occur at θ = 54° and 144°, and they are at θ = 10° and 100° for **2** (Figure 4). For both complexes, low-temperature CTM data could be reproduced by using eq 1. This implies the assumption that the ground anisotropic effective doublet is selectively populated, and the two structurally distinct paramagnetic centers have the same **g^eff^** tensor, as for EPR interpretation. Furthermore, we did not include the intramolecular interaction term determined by EPR for **2**, since its magnitude is too weak to be effective in determining CTM results.

**Figure 5:**
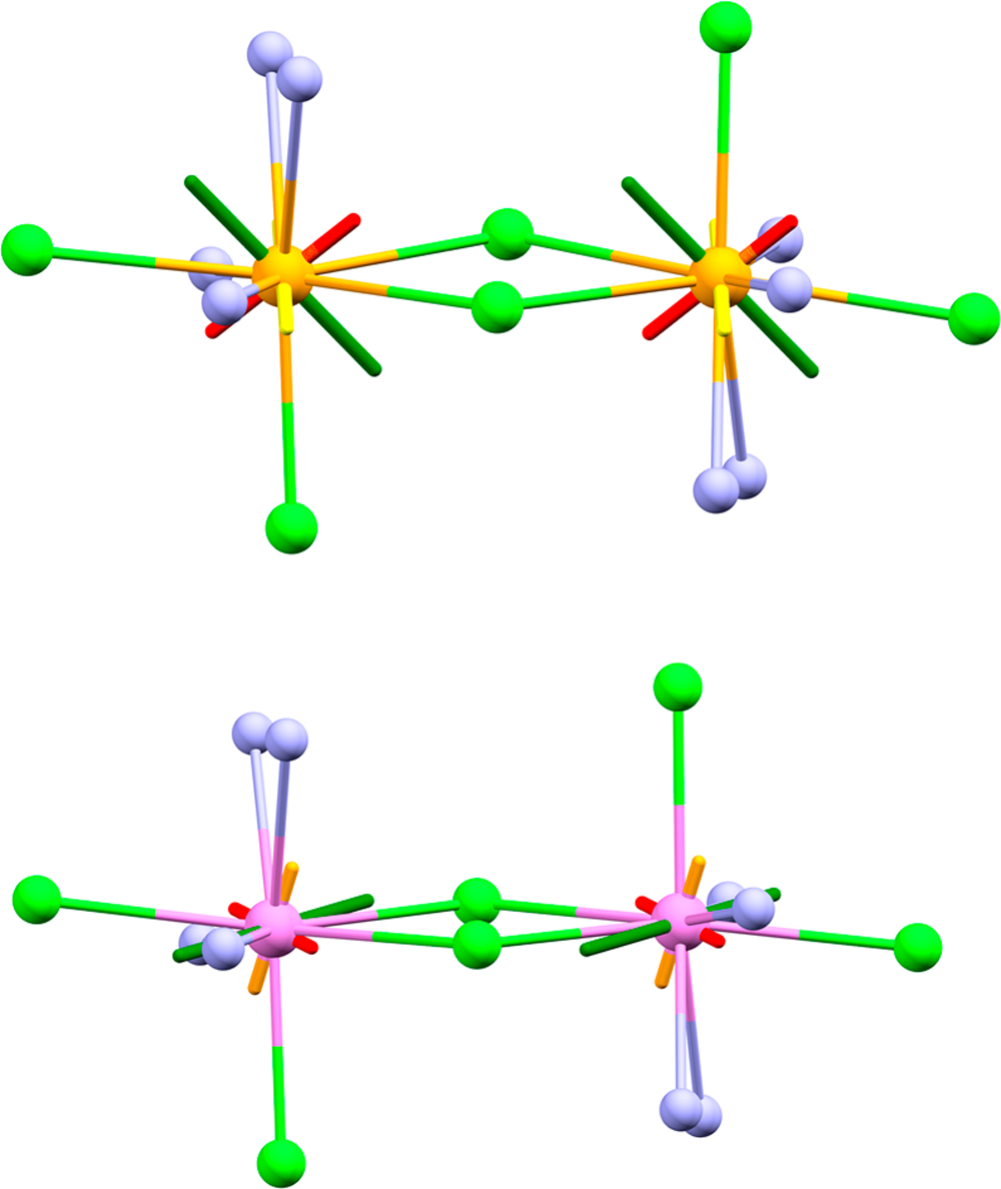
Orientation of effective principal directions of **g^eff^** tensors of the ground doublet, with respect to the molecular frame, as determined by fit of CTM data assuming collinear centers and principal *g*-values as determined by EPR for **1** (upper) and **2** (lower). Green, orange, and red rods refer to the principal components of **g^eff^** in decreasing order of magnitude; green balls represent Cl atoms, and cyan balls represent N atoms.

In the fitting procedure, the effective *g* values were kept fixed to those determined by EPR (with the exception of *g*_*z*_ for **1**, for which an upper bound of 0.5 was set), while the Euler angles (*zyz* convention) describing the orientation of **g^eff^**, with respect to the crystal frame, were left free to refine and provided best-fit results (α, β, γ) = (94.0°, 39.2°, 7.4°) and (α, β, γ) = (202.6°, 84.5°, 208.9°) for **1** and **2**, respectively. An overall scale factor was refined to account for the incertitude on the masses of the two crystals, which were too small to be determined accurately. The resulting director cosines of the easy axis, with respect to the ab′c* orthogonalized coordinate crystal system, are reported in Table S4 in the Supporting Information, while the orientation of the **g^eff^** tensors of the ground doublets, with respect to the complexes, are graphically sketched in Figure 5.

In both cases, no clear correlation between the principal directions of **g^eff^** and the structural features of the complexes emerges from the analysis of the experimental data. For **1**, the only appreciable feature is the orthogonality of the intermediate component to one Ce–Cl direction, while the major component is only very approximately oriented along the bisector of Cl–Ce–Cl. For **2**, the only evident structural correlation is the major component of the ground doublet lying in the Nd2Cl2 plane, almost orthogonal to the two long Nd–Cl bonds in this plane (95° angle).

It is interesting to note that, for **1**, the fit is of very good quality up to high temperature (see Figure S7 in the Supporting Information), thus suggesting that the ground doublet is well-isolated from the excited ones. However, since the use of the only ground state to reproduce CTM data might be an oversimplification, we turned to ab initio calculations to obtain a complete picture of the anisotropic features of these systems.

### Ab Initio Results

Multiconfigurational CASSCF calculations are the most accurate ab initio methods to rationalize the magnetic properties of lanthanide complexes.^46,47^ The widespread use of this type of calculations in this field is due to their ability to provide information that is otherwise inaccessible by most spectroscopic techniques, such as excited states’ energies and magnetic anisotropy tensors. However, they are far from being perfect and use of the results obtained by this method requires a preliminary benchmarking against a set of accurately determined properties.^17^ This approach is gaining increasing momentum in the literature: the calculated properties include low-temperature luminescence,^48,49^ EPR spectroscopy,^13,50,51^ single-crystal magnetometry,^15,52−54^ inelastic neutron scattering,^55,56^ and advanced spectroscopic techniques.^57−59^ With this aim, ab initio calculations were performed on **1** and **2**, and the results are compared with EPR and CTM data. This provides a sound basis for the following discussion on the magnetic properties of these systems.

For both complexes, CASSCF calculations predict similar but distinguishable properties for the two structurally distinct Ln^III^ centers. The energy difference between the ground and first excited doublet is above 300 cm^–1^ for both Ce^III^ centers in **1**, while for **2**, it lies at ∼40 cm^–1^ and all the ^4^I_9/2_ excited doublets are within 300 cm^–1^. In agreement with the interpretation of EPR data and with the highly distorted coordination symmetry, the calculated compositions of the ground doublets for all the paramagnetic centers are highly mixed. Indeed, all |*m*_J_⟩ components contribute by more than 5% (see Tables S5–S12 in the Supporting Information). With this electronic structure, the field-dependent magnetization curves at low temperature are correctly reproduced (see Figure S5 in the Supporting Information). As for the temperature dependence of the susceptibility, the agreement with the experimental curve is less satisfactory, suggesting some discrepancies between calculated and real energy and composition of the excited states (see Figure S4 in the Supporting Information).

For complex **1**, the computed *g*^eff^ values of the ground doublet are, on average, in very good agreement with those obtained by simulating EPR spectrum; in particular, the highest *g*^eff^ value, arbitrarily chosen as *g*_*z*_, is perfectly reproduced. The agreement with the spectral features of **2** is somehow less satisfactory, with an overestimation of the rhombicity of the ground doublet compared to the experimental EPR results, while the easy axis anisotropy is slightly underestimated. This is at variance with the overestimation of axiality commonly reported for ab initio calculations.^50^ An important point evidenced by ab initio results is the different orientation of the magnetic anisotropy axes calculated for the two centers. This offset is just 10° for **2**, but it reaches values up to 34° for the lowest *g*^eff^ components of **1** (see Table 1). Furthermore, the average orientations of the principal directions of the two centers are also quite different from the one obtained by assuming the two centers to be collinear (see Table S4 in the Supporting Information for director cosines). We checked the accuracy of the theoretical results by comparing the angular, magnetic field, and temperature dependencies of the torque calculated using ab initio results with the experimental ones. As reported in Figure S9 in the Supporting Information, the calculated CTM data for **1** are clearly offset, with respect to the experimental ones. On the other hand, for **2**, while the agreement between theoretical and experimental data is quite good for Rot2, the two are almost in phase opposition for Rot1 (see Figure S10 in the Supporting Information).

**Table 1:** Experimental EPR and Ab Initio Calculated *g*^eff^ Principal Values for the Ground Doublets of the Two Ln^III^ Centers in Cerium and Neodymium Complexes, and Angles between the Calculated Principal Orientations on the Two Sites

		EPR	**Ln1**	**Ln2**	angle **Ln1**–**Ln2**
Ce	*g*_*x*_	<0.5	0.527	0.272	34.0400
	*g*_*y*_	0.71	0.894	0.623	32.2988
	*g*_*z*_	3.0	3.065	3.066	17.9347
					
Nd	*g*_*x*_	1.08	0.586	0.743	10.3943
	*g*_*y*_	1.52	1.857	2.028	10.8398
	*g*_*z*_	4.16	3.970	3.925	9.8956

To obtain a good reproduction of the CTM data, the orientations of the anisotropy axes were then left free to be refined with respect to those calculated ab initio, while keeping the crystal field parameters (or the *g*^eff^ values of the ground doublet) fixed to the calculated values (see Figure 4, as well as Figures S6 and S7 in the Supporting Information). The best fit orientation of the **g^eff^** tensors, with respect to the ab′c* molecular frames obtained by following this procedure is reported graphically in Figure S11 in the Supporting Information and in tabular form in Table S4 in the Supporting Information.

For both derivatives, the calculated direction of the easy axis is close to that obtained by the best fit of the experimental CTM curve (angles between experimentally determined and theoretical directions ranging between 5° and 15°; see Table S13 and Figure S11 in the Supporting Information), whereas much difference is observed in the hard plane. The overestimation of rhombicity results in the intermediate and hard axes being almost swapped between calculated and experimentally derived values for Nd1 in **2** and differing by ∼50° for Nd2. Furthermore, the best fit parameters of the CTM curves are consistent with the noncollinearity of the **g^eff^** tensors of the two centers (see Table S4 and Figure S11).

To check whether this discrepancy is due to the neglecting of dynamical correlation effects on the CASSCF wave function we performed N-electron valence state perturbation theory (NEVPT2) calculations^60^ with ORCA 4.2 software^61^ (see Tables S14–S21 in the Supporting Information). However, the electronic structure description of the ground Kramers’ doublets in both compounds is not substantially modified. We can conclude that the CASSCF level of theory appears adequate and reliable for the reproduction of ground-state properties and low-temperature magnetic measurements. The discrepancies among experiment and theory should be attributed to other factors, principally the correct reproduction of the Madelung potential inside the crystal cell^62^ or the refinement of the structure by geometry optimizations.

### AC Susceptibility

Of the many lanthanide complexes presenting slow relaxation of the magnetization,^63−65^ the vast majority contains ions of the second half of the series. Dy^III^ is, by far, the most diffuse, because of its large magnetic moment and its Kramers’ nature. Ce^III^ and Nd^III^ cations can also present slow relaxation of the magnetization in zero field with a proper design of the crystal field, but only a few of them have been reported.^66−69^ For the sake of complete characterization, we measured the ac susceptibility of both **1** and **2**, to observe how the electronic structure determined above is reflected on their dynamic magnetic properties. As expected on the basis of the large transversal components of the ground doublets of these systems, the two derivatives do not show slow relaxation in zero field, because of extremely efficient QTM. However, a frequency- and temperature-dependent out-of-phase susceptibility appears on application of a dc field, more easily measurable for **2** (see Figures S12 and S13 in the Supporting Information). The temperature dependence of the relaxation times (Figure S14 in the Supporting Information) extracted by using a generalized Debye approach^70,71^ clearly points to a mix of direct and Raman contributions rather than to an Orbach process for both derivatives.^72^ This is again consistent with the largely rhombic nature of these systems indicated by EPR and CTM experiments and with the absence of a reliable anisotropy barrier evidenced by ab initio calculations. In this respect, despite **1** and **2** being part of the scarce number of Ce^III^ and Nd^III^ complexes reported until now that present slow relaxation of the magnetization under certain conditions,^42,50,66−69,73−75^ we cannot consider them as SMM. Indeed, neither a value for an effective anisotropy barrier *U*_eff_ nor a blocking temperature can be defined for these complexes.^3^ It is also interesting to note that for **2**, two relaxation processes are active. While this might be, in principle, attributed to the two different single-ion properties, their largely different values rather suggest that the slowest of the two is to be attributed to a collective process. However, given the small number of points and the impossibility of obtaining an isostructural diamagnetic analogue, we refrain here from drawing any further conclusions from these data.

## Discussion

The above-reported results point to the absence of evident correlation between the coordination sphere of the lanthanides in the two complexes and the orientation of the magnetic anisotropy axes at low temperature. This is to be attributed to the absence of a pronounced axial charge distribution of the ligand environment around the lanthanide center, which would favor pure easy axis anisotropy for both Ce^III^ and Nd^III^, oblate ions,^76^ in agreement with the largely distorted coordination polyhedra.

The comparison of the experimental and theoretical results further indicates that, while ab initio calculations work remarkably well in determining the orientation of the easy magnetization directions of the lanthanide centers, it does not provide results that are as reliable as those for the orientation of the hard directions. The same is true for the accuracy of the reproduction of the principal values, which is highest for the largest *g*^eff^ values but decreases for the hardest directions. However, the most relevant result obtained by ab initio methods is for sure the indication that the **g^eff^** tensors of the two centers are far from being collinear. In this respect, our previous analysis of the EPR spectrum, based on collinearity assumption, is somehow oversimplified. A refinement of the model would then require performing simulations on the basis of the following Spin Hamiltonian:^77^
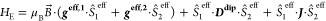
3Here, **g**^**eff,*i***^ are the *g*-tensors expressed in the molecular reference frame, which were fixed at the principal *g*^eff^ values obtained by preliminary EPR interpretation and principal directions obtained by the CTM fits based on ab initio results; **D^dip^** is the dipolar interaction tensor, which can be calculated using a point dipole approximation; **J** represents the exchange interaction tensor, which, in principle, has no symmetry and can be decomposed into antisymmetric, anisotropic, and isotropic components.^78^ For **1**, the simple inclusion of the noncollinearity of **g**^**eff**^ tensors on the two sites and of the calculated dipolar tensor, with **J** constrained to be isotropic with an absolute value smaller than 0.02 cm^–1^ provides a clear improvement with respect to the original simulation (Figure 2). This set of parameters is indeed able to partially reproduce the series of weak signals which might otherwise have been misassigned as a nonstatistical distribution of the microcrystals.

The situation is more complex for **2**, given the large noncollinearity of the two tensors indicated by ab initio calculations and CTM, and the large discrepancy between the two sets of calculated and experimental results (see Figure S10 in the Supporting Information). At any rate, by considering a noncollinearity of the single ion **g^eff^**, inclusion of the calculated dipolar interaction and of an isotropic exchange coupling is not enough to provide a reasonable simulation of the multifrequency EPR spectra, thus confirming the relevance of the anisotropic component of the interaction. As a whole, this analysis confirms that the intramolecular anisotropic exchange coupling interactions are larger in **2** than in **1**, being however small, in terms of absolute values.

To the best of our knowledge, these are the first (di)chloro-bridged Ce^III^ and Nd^III^ dimers structurally characterized for which an in-depth analysis of the magnetic properties has been performed. Previous reports were indeed only of qualitative nature and did not provide any hint as to the intramolecular magnetic interactions in these systems.^79,80^ More-detailed studies have been reported for dichloro-bridged heavy lanthanide complexes (Dy- and Er-based), given their interest as SMM^81−86^ and, more recently, for other halogen-bridged complexes.^87,88^ In those systems, the large easy-axis anisotropy of the single-ion, which is a prerequisite for observing SMM behavior, provides a much stronger dipolar interaction (more than one order of magnitude) than in the present case. At the same time, the exchange contribution, either ferromagnetic or antiferromagnetic, turned out to be weak but crucial in determining specific features of the magnetization dynamics. The situation is evidently much different for **1** and **2**. Here, the single-ion rhombic anisotropy hampers SMM behavior and the dipolar interaction is weak and oriented along a direction which requires single-crystal experimental study to be determined.

## Conclusions

Here, we have reported an in-depth study of the single-ion anisotropy and intramolecular magnetic interactions for two chloro-bridged dinuclear lanthanide complexes. EPR, CTM, and ab initio calculations, for both systems, point to a clearly rhombic magnetic anisotropy, the orientation of which has no direct relation to structural features. EPR spectroscopy clearly indicates that intramolecular interactions for the cerium dimer, **1**, are weak and essentially of a dipolar nature, whereas for the neodymium dimer, **2**, anisotropic exchange provides a major contribution. From a methodological point of view, this study demonstrates that, in the case of largely rhombic systems, ab initio calculations are still reliable in determining the principal values of the anisotropy tensors but can fail in determining the orientation of their weakest component. In turn, however, the result obtained by ab initio methods may help to fix some of the unknowns in the analysis of EPR and CTM results. The combination of all these techniques appears of fundamental importance to unravel the determination of the weak intramolecular exchange interactions characterizing lanthanide-based polynuclear complexes, which play a subtle but relevant role in determining the magnetic properties of these systems.

## Experimental Section

### Synthesis

All the starting materials were purchased from TCI and were used without further purification.

The **L1** ligand resulted from the condensation of 2-pyridinecarboxaldehyde (0.053 g, 0.5 mmol) and 1*R*,2*R*-1,2-diphenylethylendiamine (0.053 g, 0.25 mmol) in 20 mL of methanol stirred at room temperature for 1 h.

Ligand was used *in situ* for the preparation of the complexes.

All the complexes were prepared following the same procedure. The previously prepared ligand solution was added to an equimolar quantity of the corresponding lanthanide chloride, LnCl_3_**·***x*H_2_O (0.25 mmol, *x* = 7, 0.093 g for Ce, *x* = 6, 0.098 g for Nd, *x* = 6, 0.91 g for Sm), dissolved in 10 mL of methanol. The resulting solution was stirred at room temperature for 1 h. Orange (**1**) and white (**2** and **3**) crystals suitable for XRD were obtained from slow diffusion of the solution in diethyl ether, with a yield of ∼30% for all the complexes. These were used for the instrumental measurements. Calculated/found elemental analysis (dried samples) were as follows: for **1**: C, 49.03/49.2; N, 8.80/8.8; H, 3.48/3.3%; for **2**: C, 48.71/48.6; N, 8.74/8.7; H, 3.46/3.7%; and for **3**: C, 48.25/48.4; N, 8.66/8.9; H, 3.43/3.6%.

### X-ray Crystallography

Prismatic crystals of **1** and **2** were used for single-crystal X-ray crystallographic analysis. Measurements were performed after exposing **3** for 24 h to ambient conditions, resulting in **3b**. The X-ray intensity data were collected on a D8 Venture system equipped with a multilayer monochromator and a Mo microfocus (λ = 0.71073 Å). The frames were integrated with the Bruker SAINT software package, using a narrow-frame algorithm. Data were corrected for absorption effects using the multiscan method (SADABS). The structures were solved and refined using the Bruker SHELXTL software package. Further crystallographic details can be found in the corresponding CIF files provided in the Supporting Information.

Indexation for monocrystal studies was performed in a SCD Oxford Xcalibur3 X-ray diffractometer.

Powder XRD spectra were acquired with a PANalytical X’Pert PRO MPD θ/θ powder diffractometer of 240 mm of radius in a configuration of convergent beam with a focalizing mirror and transmission geometry with flat samples sandwiched between low absorbing films and Cu Κα radiation (λ = 1.5418 Å). Comparison between the calculated spectrum from the single-crystal structures of **3b** is an accurate match with the experimental powder diffraction of **1**, **2**, and **3**.

### Physical Characterization

All the physical measurements on single crystals were performed on fresh samples corresponding to the solvated structures. Measurements on powders should be considered as being performed on desolvated structure (see the section on Structural description).

#### EPR Spectroscopy

X-band (∼9 GHz) and W-band (∼94 GHz) EPR spectra were recorded using commercial Bruker E500 and E600 spectrometers. The samples were microcrystalline ground powders, undiluted. For W-band spectra, sample was embedded in wax to avoid torquing effects. Cryogenic temperatures were obtained by using a CF935 continuous flow cryostat for W-band spectrometer and a ESR900 for X-band spectrometer, both from Oxford Instruments. Simulation of the spectra was performed using the Matlab toolbox Easyspin.^89^

#### Cantilever torque magnetometry

CTM measurements were performed by using a homemade two-legged CuBe cantilever separated by 0.1 mm from a gold plate. The cantilever was inserted into an Oxford Instruments MAGLAB2000 platform with automated rotation of the cantilever chip in a vertical magnet. The capacitance of the cantilever was detected with an Andeen-Hegerling 2500A Ultra Precision Capacitance Bridge. The faces of the measured crystal were indexed by using XRD in the above-described setup. This procedure resulted in an estimated uncertainty of the actual orientation of the crystal of ∼5°.^90^ The fit of the data was performed using home-developed codes based either on FORTRAN^44^ or on MATLAB exploiting EasySpin toolbox.^91^

#### Magnetic Measurements

Magnetic measurements were performed using a Quantum Design MPMS SQUID magnetometer and the dynamic measurements were performed in a Quantum Design PPMS in the ac mode on powders pressed in a pellet to avoid field-induced orientations. Diamagnetic corrections were calculated using Pascal’s constants.^92^

#### Electronic Circular Dichroism

Electronic circular dichroism spectra were recorded in methanolic solutions on a JASCO Model 815 spectropolarimeter.

### Computational Details

All calculations have been performed with the MOLCAS 8.0 quantum chemistry package.^93^ The geometries resolved by XRD were employed without further geometry optimizations. The calculations for each ion in each dimer were performed by substituting the second lanthanide ion in the molecule with its diamagnetic equivalent, lanthanum. The energy ladder of the electronic states for every lanthanide ion has been computed within the Complete Active Space Self Consistent Field approach, followed by Spin Orbit State Interaction calculation (the CASSCF/CASSI-SO method).^94^ The employed ANO-RCC basis sets^95,96^ and contractions are reported in Table S22 in the Supporting Information. The chosen active space for the lanthanides consists of the unpaired electrons in the seven 4f -orbitals of the lanthanide ion in the oxidation state +3: CAS (1,7) for Ce and CAS (3,7) for Nd, respectively. Because of hardware limitations, only the states with the highest spin multiplicity for each lanthanide were included in the following CASSI-SO calculation: 7 doublets for Ce, 35 quadruplets for Nd. The **g^eff^** for each Kramers’ doublet were computed with the SINGLE_ANISO module.^62^ The magnetic anisotropy was investigated within the pseudospin framework and their anisotropy axes were calculated with a pseudospin *S* = 1/2.

## Supporting Information Available

ECD spectrum, PXRD spectra, further crystallographic figures and crystallographic tabular materials, dc and ac magnetic characterization curves, full CTM measurements, ab initio calculated Stevens’ parameters for the four paramagnetic centers, orientations of the anisotropy axis for different models, in both tabular and graphic mode (PDF)

## Accession Codes

CCDC 2057194–2057197 contain the supplementary crystallographic data for this paper. These data can be obtained free of charge via www.ccdc.cam.ac.uk/data_request/cif, or by emailing data_request@ccdc.cam.ac.uk, or by contacting The Cambridge Crystallographic Data Centre, 12 Union Road, Cambridge CB2 1EZ, UK; fax: +44 1223 336033.

## ABBREVIATIONS

CTM: cantilever torque magnetimetry
cw-EPR: continuous wave electron paramagnetic resonance
CASSCF: complete active space self consistent field
