# Supplementary material for: Single-Ion Anisotropy and Intramolecular Interactions in CeIII and NdIII Dimers
Source: Inorg Chem. 2021 Jun 10;60(12):8692–703. doi: 10.1021/acs.inorgchem.1c00647 (PMC8277162; doi:10.1021/acs.inorgchem.1c00647)
Supplement: Supplementary file 1 — ic1c00647_si_001.pdf [file ic1c00647_si_001.pdf]

Supporting Information

Single ion anisotropy and intramolecular interactions in Ce^{III} and Nd^{III} dimers

Julia Mayans[¥,§], Lorenzo Tesi[¶,†], Matteo Briganti[¶,⊥], Marie-Emmanuelle Boulon,[¶] Mercè Font-Bardia,[▽] Albert Escuer[¥] and Lorenzo Sorace[¶,*]

[¥]Departament de Química Inorgànica i Orgànica, Secció Inorgànica and Institute of Nanoscience and Nanotechnology (IN^2UB), Universitat de Barcelona. Martí i Franques 1-11, Barcelona-08028, Spain.

[¶] Dipartimento di Chimica "Ugo Schiff" & INSTM RU, Università degli Studi di Firenze, Via della Lastruccia 3-13, 50019 Sesto Fiorentino (Firenze), Italy.

[▽]Unitat de Difracció de R-X, Centre Científic i Tecnològic de la Universitat de Barcelona (CCiTUB), Universitat de Barcelona, Solé i Sabarís 1-3, 08028 Barcelona, Spain.

Present address:

§ Instituto de Ciencia Molecular (ICMol), University of Valencia. Catedrático José Beltran 2, 46980 Paterna (Valencia), Spain.

† Institute of Physical Chemistry, University of Stuttgart, Pfaffenwaldring 55, 70569 Stuttgart, Germany.

⊥ Department of Chemistry, Federal University of Parana, Centro Politecnico, Jardim das Americas, 81530-900 Curitiba-PR, Brazil

* e-mail: lorenzo.sorace@unifi.it

ECD spectra

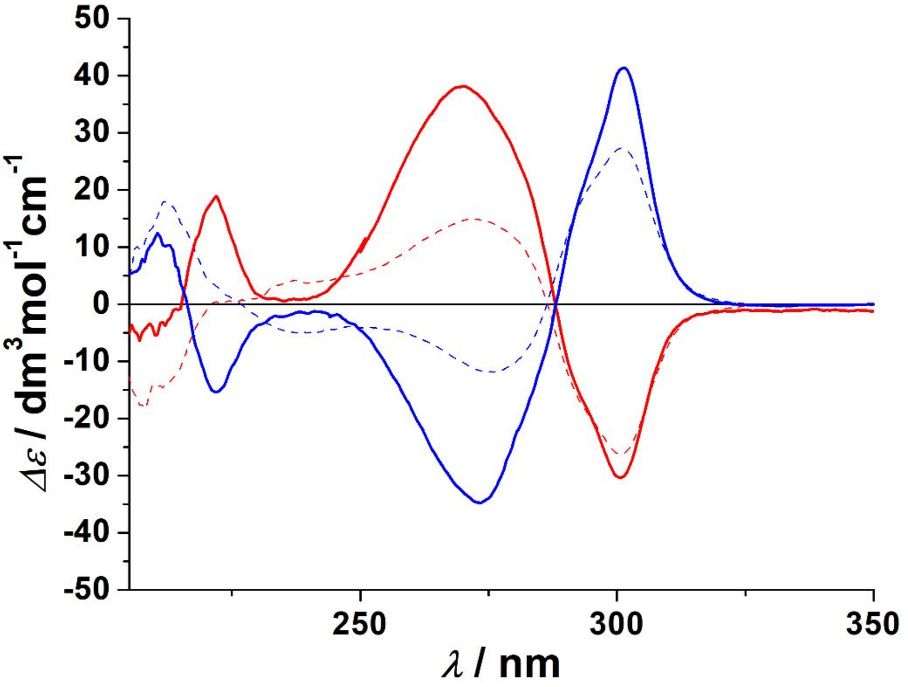

Figure S1. Electronic circular dichroism spectra for complexes **2*RR*** (solid red line) and **2*SS*** (solid blue line), measured in methanolic solution. The dashed lines correspond to the related monomeric complexes [Ln(L)Cl_3] previously reported (main text ref. 37). Its different shape at low wavelengths allows to differentiate the mono and dinuclear systems, confirming its stability in solution.

X-Ray diffraction

Table S1. Crystal data and structure refinement parameters for fresh crystals of compounds **1*RR***, **2*RR***, **2*SS*** and **3b,** obtained by leaving **3** 24 hours air-exposed

	**1*RR***	**2*RR***	**2*SS***	**3b**
Formula	$C_{52}H_{47}Ce_2Cl_6N_8O_{1.5}$	$C_{53}H_{51}Nd_2Cl_6N_8O_{2.5}$	$C_{52.5}H_{47.5}Nd_2Cl_6N_8O_{1.25}$	$C_{52}H_{44}Sm_2Cl_6N_8$
FW	1300.91	1341.20	1311.66	1294.35
System	Triclinic	Triclinic	Triclinic	Triclinic
Space group	P 1	P 1	P 1	P1
a/Å	9.734(1)	9.7069(4)	9.7140(6)	9.707(1)
b/Å	11.195(1)	11.1708(5)	11.1726(6)	11.853(1)
c/Å	15.216(2)	15.1938(7)	15.1372(9)	12.593(1)
α/°	73.237(5)	73.354(2)	73.447(2)	86.873(4)
β/°	77.827(5)	77.880(1)	77.847(2)	70.515(3)
γ/°	64.447(4)	64.663(1)	64.911(2)	73.949(3)
V/ Å^3	1425.1(3)	1419.4(1)	1418.6(2)	1311.6(2)
Z	1	1	1	1
T, K	100(2)	100(2)	105(2)	299(2)
$\lambda(MoK_\alpha)$, Å	0.71073	0.71073	0.71073	0.71073
ρ_{calc}, g·cm^{-3}	1.516	1.569	1.535	1.639
$\mu(MoK_\alpha)$, mm^{-1}	1.902	2.138	2.136	2.566
Flack param.	0.14(2)	0.01(2)	0.08(2)	0.031(7)
R	0.0396	0.0298	0.0293	0.0193
ωR^2	0.1097	0.0708	0.0843	0.0409

Table S2. Selected bond distances (Å) and angles (deg) for complexes **1*RR***, **2*RR***, **2*SS*** and **3b.**

	1RR	***2RR***	***2SS***	**3b**
**Ln1-N1**	2.574(12)	2.661(10)	2.609(9)	2.66(1)
**Ln1-N2**	2.614(10)	2.543(9)	2.604(7)	2.58(2)
**Ln1-N3**	2.638(11)	2.588(8)	2.559(10)	2.53(2)
**Ln1-N4**	2.691(12)	2.597(9)	2.667(10)	2.56(2)
**Ln1-Cl1**	2.721(4)	2.805(3)	2.800(3)	2.772(6)
**Ln1-Cl2**	2768(4)	2.892(2)	2.898(2)	2.889(7)
**Ln1-Cl3**	2.840(3)	2.701(3)	2.702(3)	2.689(5)
**Ln1-Cl4**	2.903(3)	2.741(3)	2.747(3)	2.684(6)
**Ln2-N5**	2.703(12)	2.676(9)	2.678(9)	2.60(1)
**Ln2-N6**	2.643(11)	2.597(9)	2.576(9)	2.50(2)
**Ln2-N7**	2.618(10)	2.563(8)	2.547(8)	2.53(2)
**Ln2-N8**	2.589(13)	2.584(10)	2.581(10)	2.60(2)
**Ln2-Cl1**	2.931(3)	2.902(3)	2.903(3)	2.923(7)
**Ln2-Cl2**	2.845(3)	2.808(3)	2.816(3)	2.813(6)
**Ln2-Cl5**	2.767(4)	2.702(3)	2.705(3)	2.671(5)
**Ln2-Cl6**	2.747(3)	2.740(3)	2.741(3)	2.697(6)
**Ln1-Cl1-Ln2**	109.1(1)	109.65(9)	109.92(9)	111.2(2)
**Ln1-Cl2-Ln2**	109.8(1)	109.86(9)	109.62(9)	111.0(2)

Table S3. Continuous Shape Measurements analysis of the coordination polyhedra around lanthanide ions in **1** and **2** and best fit agreement obtained using SHAPE. Bold fonts highlight the best agreement.

	**SAPR-8**	**TDD-8**	**JBTPR-8**	**BTPR-8**
**Ce1**	3.893	3.282	3.915	**2.798**
**Ce2**	4.182	**2.469**	4.353	3.268
**Nd1**	3.914	3.198	3.880	**2.794**
**Nd2**	3.544	**2.156**	3.819	2.814

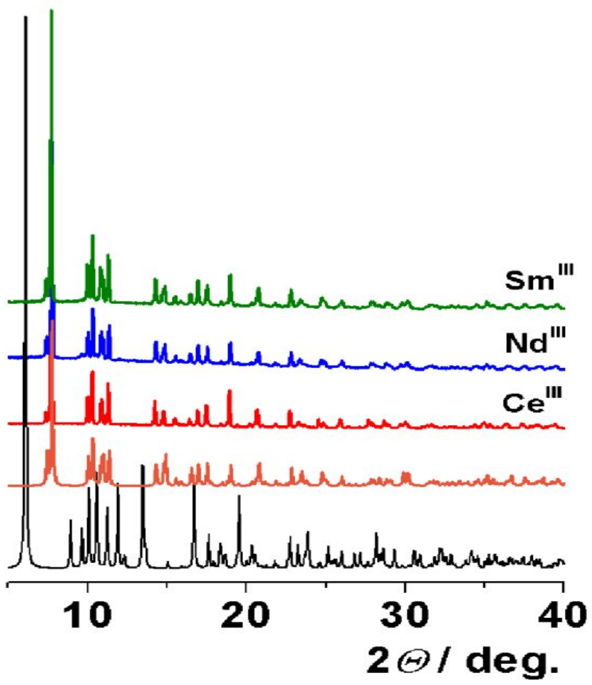

Figure S2. Experimental powder X-ray diffraction spectra of isostructural Ce^{III}, **1** Nd^{III}, **2**, and Sm^{III}, **3** derivatives compared with the simulated spectrum obtained by the crystal structure of the complex **2**, containing crystallization solvent molecules (black trace) and that obtained by the crystal structure of the desolvated Sm^{III} derivative **3b** (orange trace).

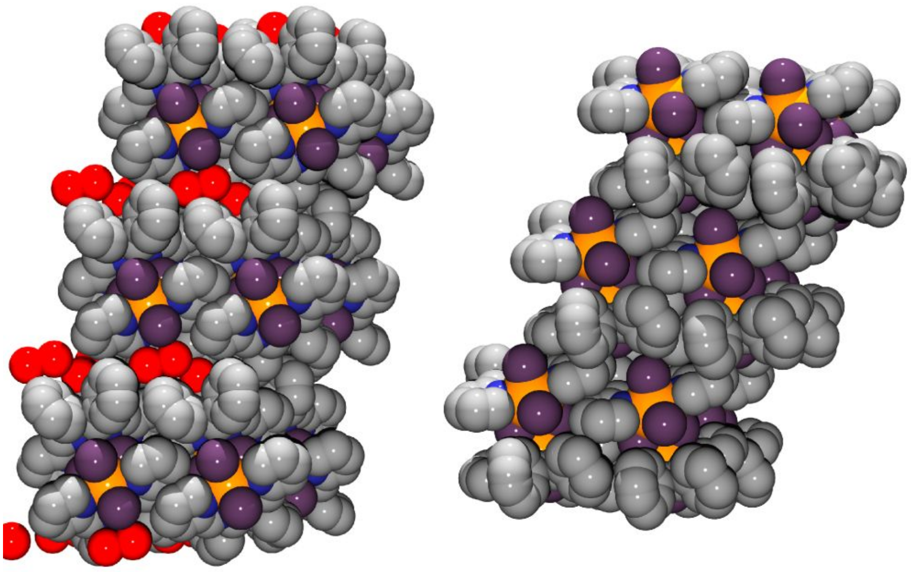

Figure S3. Spacefill plot of the structures of **2** (left) showing the layers of the crystallization molecules in the *ab* plane of the cell and structure of **3b** (right) showing the compacted structure.

Dc Magnetic characterization

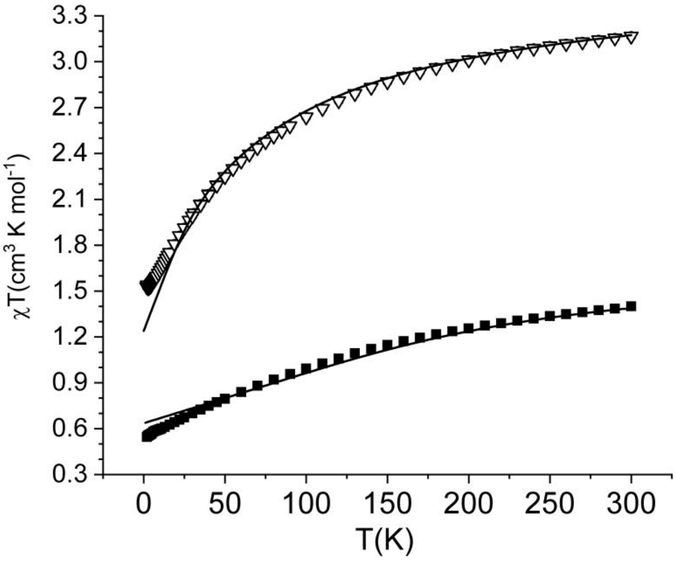

Figure S4 $\chi_M T$ vs. T for polycrystalline powder samples of **1** (squares) and **2** (triangles). The solid lines represent the simulated curves obtained by ab initio calculations.

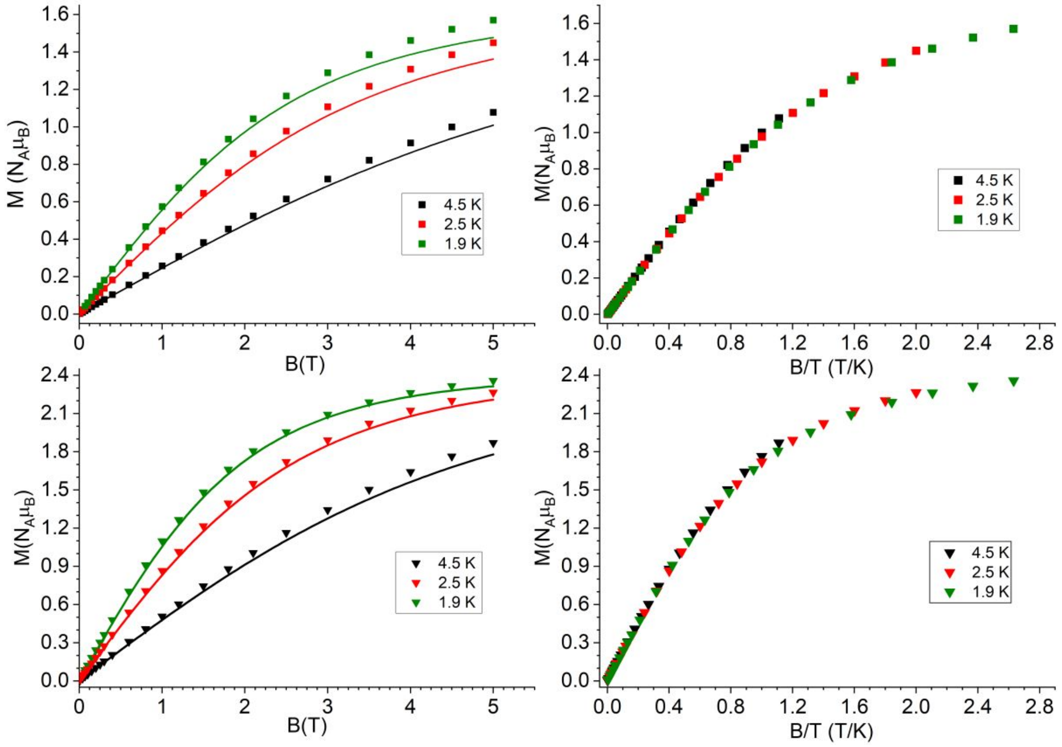

Figure S5. Isothermal magnetization (left panels) and reduced magnetization (right panels) for polycrystalline powder samples of **1** (squares, upper panels), and **2** (triangles, lower panels), measured at three different temperatures (see legends). The solid lines represent the simulated curves obtained by ab initio calculations.

Cantilever torque magnetometry (CTM)

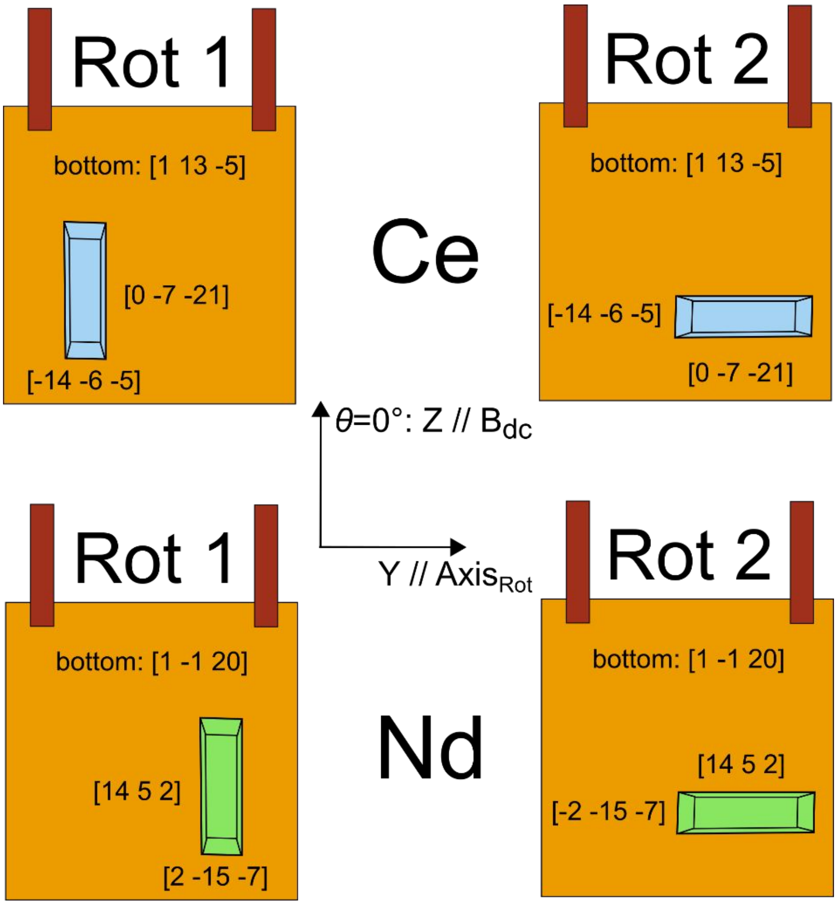

Figure S6. Schematic representation of the orientation of crystals of **1** (upper) and **2** (lower) on CTM with respect to the laboratory frame in the two rotations (Rot1 and Rot2). The orientations are given as [h k l] indexes as taken from X-Ray diffractometer experiments. The crystals are placed on the CTM cantilever (orange square) according to the starting position with respect to the laboratory reference frame (XYZ).

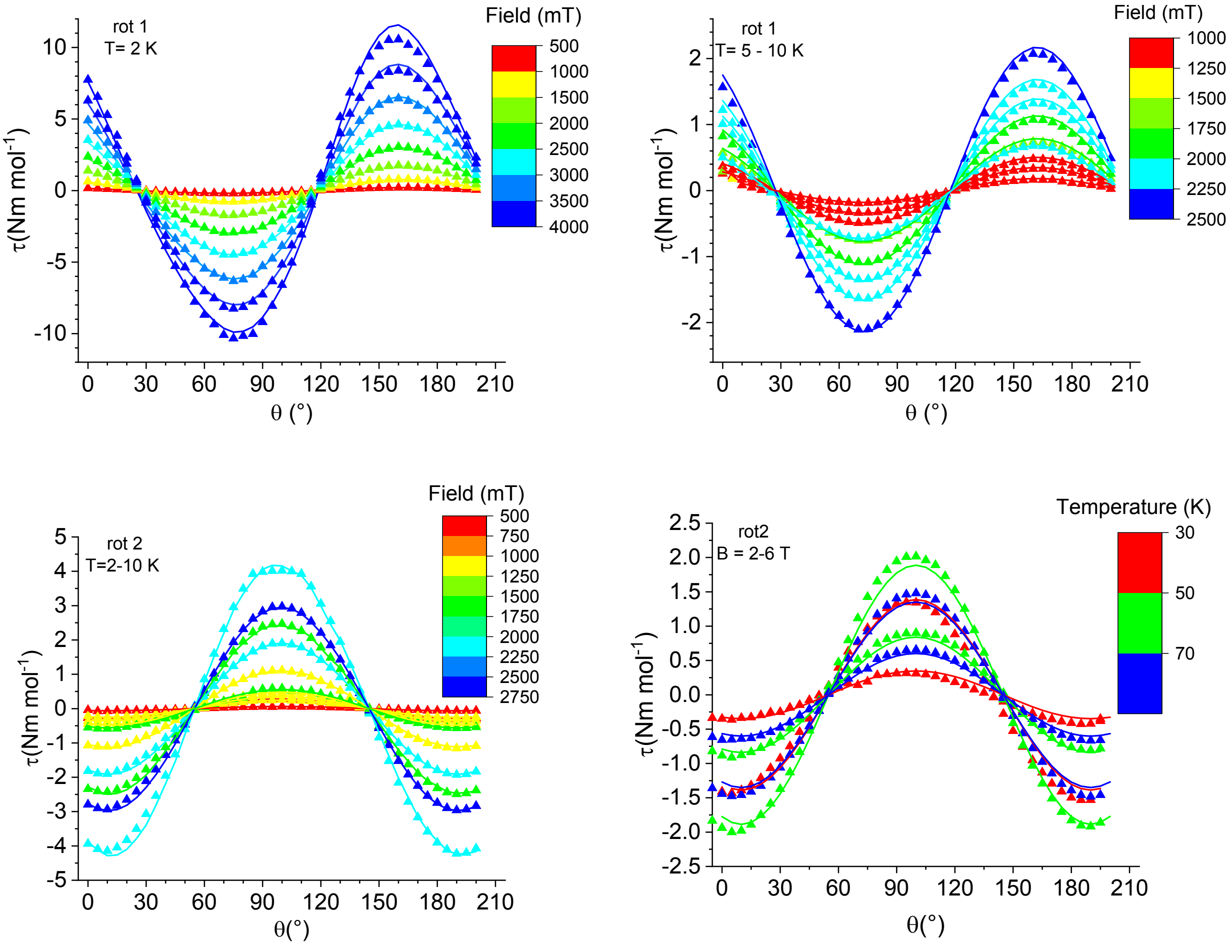

Figure S7. Experimental and fitted CTM data for **1** at variable temperatures and fields. Panels on first line: first rotation; Panels on second line: second rotation. The fitted curves are obtained by using the ab initio calculated values of the ground doublet g tensors for the two centers and leaving their orientation free to refine. Best fit parameters are reported in Table S4.

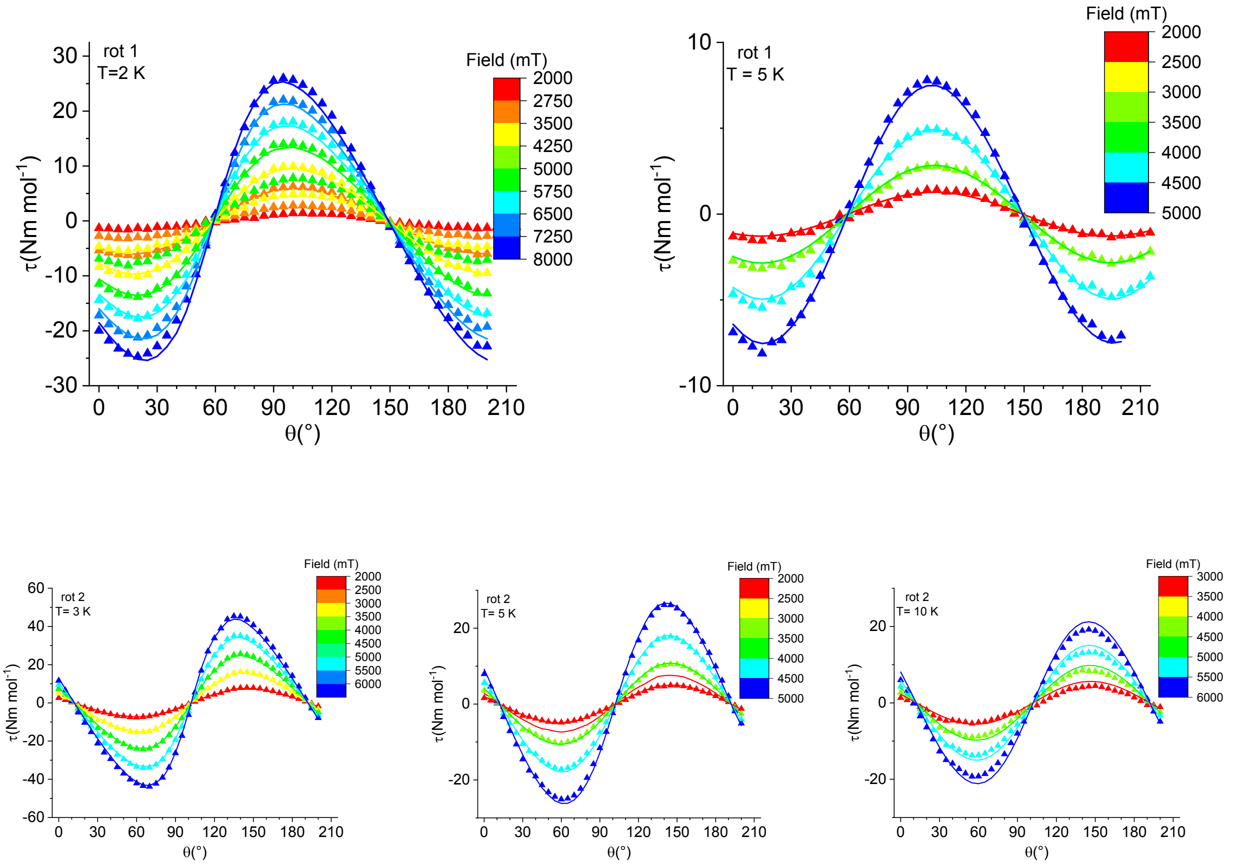

Figure S8. Experimental and fitted CTM data for **2** at variable temperatures and fields. Upper panel: first rotation; lower panels: second rotation. The fitted curves are obtained by using the ab initio calculated values of the ground doublet g tensors for the two centres and leaving their orientation free to refine. Best fit parameters are reported in Table S4.

Table S4 Director cosines of the ground tensor g doublets with respect to the *ab'c** orthogonalized reference system, as obtained by the fit of CTM data. When considering a single center, the principal g values were fixed to the EPR derived ones (with g_x=0.4 for **1**). When considering two centers, the principal g values were fixed to the ab initio derived ones.

**Center**	**Principal g value**	**Orientations of principal g values**		
		a	*b'*	*c**
**Ce_{ave}**	0.4	-0.1491	-0.9887	-0.0187
	0.71	0.7650	-0.1273	0.6313
	3.0	-0.6265	0.0798	0.7753
**Ce1**	0.527	-0.6615	-0.4766	-0.5790
	0.894	0.3571	-0.8791	0.3156
	3.065	-0.6595	0.002	0.7517
**Ce2**	0.272	0.6474	0.67395	0.3558
	0.623	-0.45206	0.7155	-0.5326
	3.066	-0.6136	0.1840	0.7679
**Nd_{ave}**	1.08	-0.0225	0.3306	0.9435
	1.52	-0.3063	-0.9006	0.3083
	4.16	0.9517	-0.2821	0.1216
**Nd1**	0.586	0.2132	0.5213	0.8263
	1.857	-0.3272	-0.7588	0.5631
	3.970	0.9206	-0.3905	0.0087
**Nd2**	0.743	-0.1767	0.1587	0.9714
	2.028	-0.3236	-0.9414	0.0949
	3.925	0.9295	-0.2975	0.2177

Ab initio CASSCF calculations with MOLCAS

Table S5. Ab initio calculated g-tensor main values and orientations of the main magnetic axes in the crystallographic frame for the three lowest lying Kramers' doublets of **Ce1** in **1**, corresponding to the ground atomic multiplet J = 5/2.

**Energy (cm^{-1})**	***g*-tensor**		**Orientations of main magnetic axes**		
		Main Values	*a*	*b'*	*c**
0	g_x	0.527	-0.539549	-0.813079	-0.218609
	g_y	0.894	0.735706	-0.581551	0.347182
	g_z	3.065	-0.409418	0.026490	0.911962
348	g_x	0.473	-0.293622	-0.466740	-0.834231
	g_y	0.543	0.936911	-0.313692	-0.154256
	g_z	3.522	-0.189695	-0.826893	0.529400
432	g_x	0.507	-0.079311	-0.389990	0.917397
	g_y	0.963	0.964965	-0.260935	-0.027501
	g_z	3.472	0.250106	0.883075	0.397021

Table S6. Ab initio calculated g-tensor main values and orientations of the main magnetic axes in the crystallographic frame for the three lowest lying Kramers'doublets of **Ce2** in **1**, corresponding to the ground atomic multiplet J = 5/2

Energy (cm^{-1})	***g***-tensor		Orientations of main magnetic axes		
		Main Values	*a*	*b'*	*c**
0	g_x	0.272	-0.680434	-0.403074	-0.611997
	g_y	0.623	0.296825	-0.915159	0.272726
	g_z	3.066	-0.670003	0.003916	0.742348
320	g_x	0.545	0.993561	-0.033923	0.108100
	g_y	1.066	-0.079376	0.472403	0.877801
	g_z	3.615	-0.080844	-0.880730	0.466669
411	g_x	1.164	0.239137	-0.488954	0.838891
	g_y	1.595	0.817849	0.567128	0.097416
	g_z	2.296	-0.523390	0.662790	0.535511

Table S7. Calculated crystal field parameters, expressed as Extended Stevens'Operators (ESO) coefficients, of the ground atomic multiplet J = 5/2 for **Ce1** and **Ce2** in **1**.

**ESO Coefficients**	Value (cm^{-1})	
	**Ce1**	**Ce2**
B_2^{-2}	-13.2	-4.78
B_2^{-1}	-2.51	-11.1
B_2^{0}	-10.1	-10.1
B_2^{1}	2.45	-6.50
B_2^{2}	14.9	-5.47
B_4^{-4}	-0.259	0.136
B_4^{-3}	-1.53	4.15
B_4^{-2}	-3.65	-1.12
B_4^{-1}	-1.77	-3.48
B_4^{0}	-0.440	-0.583
B_4^{1}	3.05	0.492
B_4^{2}	1.46	-3.35
B_4^{3}	0.875	4.43
B_4^{4}	-0.476	0.417

Table S8. Decomposition of the three lowest lying Kramers' doublets, corresponding to the ground atomic multiplet J = 5/2, in wavefunctions with definite projections of the total moment J, for the two paramagnetic centers of complex **1** as calculated ab initio.

	**Kramers'Doublets Energies (cm^{-1})**	**Composition of the Kramers'doublets (%)**		
		**|±5/2 >**	**|±3/2 >**	**|±1/2 >**
**Ce1**	0	64.6	5.9	29.6
	348	24.2	56.2	19.5
	432	11.2	37.9	50.9
**Ce2**	0	66.8	7.0	26.2
	320	31.5	14.8	53.8
	411	1.7	78.2	20.1

Table S9. Ab initio calculated *g*-tensor main values and orientations of the main magnetic axes in the crystallographic frame for the five lowest lying Kramers'doublets of **Nd1** in **2**, corresponding to the ground atomic multiplet J = 9/2.

Energy (cm^{-1})	***g*-tensor**		Orientations of main magnetic axes		
		Main Values	*a*	*b'*	*c**
0	g_x	0.586	-0.261453	-0.713044	0.650547
	g_y	1.857	-0.208554	-0.616337	-0.759364
	g_z	3.970	0.942416	-0.334212	0.012435
37	g_x	0.359	-0.259665	-0.652159	-0.712224
	g_y	1.270	0.820177	-0.538268	0.193851
	g_z	4.348	-0.509789	-0.533814	0.674654
85	g_x	0.010	-0.015917	-0.596832	-0.802208
	g_y	0.675	0.992118	0.090306	-0.086871
	g_z	3.149	0.124292	-0.797268	0.590690
226	g_x	1.162	-0.219344	-0.697587	-0.682100
	g_y	1.496	0.932546	0.055602	-0.356745
	g_z	2.812	0.286787	-0.714339	0.638336
313	g_x	0.061	-0.396170	-0.308998	-0.864621
	g_y	1.311	0.802116	-0.574737	-0.162131
	g_z	4.302	-0.446832	-0.757758	0.475546

Table S10. Ab initio calculated g-tensor main values and orientations of the main magnetic axes in the crystallographic frame for the five lowest lying Kramers'doublets of **Nd2** in **2**, corresponding to the ground atomic multiplet J = 9/2.

Energy (cm^{-1})	***g*-tensor**		Orientations of main magnetic axes		
		Main Values	*a*	*b'*	*c**
0	g_x	0.743	-0.126536	-0.647058	0.751868
	g_y	2.028	-0.111848	-0.743820	-0.658955
	g_z	3.925	0.985636	-0.167477	0.021748
40	g_x	0.081	-0.270918	-0.605687	-0.748163
	g_y	0.988	0.839541	-0.528914	0.124184
	g_z	4.534	-0.470930	-0.594469	0.651791
77	g_x	0.052	-0.527196	0.503269	0.684679
	g_y	0.933	-0.836748	-0.167083	-0.521474
	g_z	3.299	-0.148043	-0.847823	0.509196
214	g_x	0.066	0.715378	0.543992	0.438528
	g_y	1.350	-0.691564	0.461531	0.555633
	g_z	2.733	0.099866	-0.700758	0.706374
293	g_x	0.396	-0.315641	-0.432396	-0.844633
	g_y	1.232	0.881952	-0.462065	-0.093040
	g_z	4.127	-0.350045	-0.774293	0.527200

Table S11. Ab initio calculated crystal field parameters, expressed as Extended Stevens'Operators (ESO) coefficients, of the ground atomic multiplet J = 9/2 for **Nd1** and **Nd2** in **2**

**ESO Coefficients**	Value (cm^{-1})	
	**Nd1**	**Nd2**
B_2^{-2}	-2.15	-1.74
B_2^{-1}	0.834	0.721
B_2^{0}	-0.202	0.00515
B_2^{1}	1.53	2.00
B_2^{2}	-0.106	-0.261
B_4^{-4}	-0.0709	-0.00450
B_4^{-3}	-0.141	-0.218
B_4^{-2}	0.0209	0.0202
B_4^{-1}	-0.00225	-0.0165
B_4^{0}	0.000480	0.00214
B_4^{1}	-0.0521	-0.0839
B_4^{2}	0.0677	0.0461
B_4^{3}	0.120	0.165
B_4^{4}	-0.139	-0.135
B_6^{-6}	-0.00242	-0.00497
B_6^{-5}	-0.0316	-0.0435
B_6^{-4}	-0.00549	0.00367
B_6^{-3}	0.00657	0.00356
B_6^{-2}	0.00228	-0.00337
B_6^{-1}	-0.00548	-0.00531
B_6^{0}	-0.000643	-0.000598
B_6^{1}	-0.00248	-0.0115
B_6^{2}	0.0103	0.00439
B_6^{3}	-0.000558	0.00810
B_6^{4}	-0.00570	-0.00575
B_6^{5}	0.0458	-0.00124
B_6^{6}	0.00135	0.000123

Table S12. Decomposition of the five lowest lying Kramers' doublets, corresponding to the ground atomic multiplet J = 9/2, in wavefunctions with definite projections of the total moment J, for the two paramagnetic centers of complex **2** as calculated ab initio.

	**Kramers'Doublets Energies (cm^{-1})**	**Composition of the Kramers'doublets (%)**				
		**|±9/2 >**	**|±7/2 >**	**|±5/2 >**	**|±3/2 >**	**|±1/2 >**
**Nd1**	0	43.1	7.6	25.5	12.4	11.4
	37	10.4	24.6	24.1	9.9	30.9
	85	9.7	6.3	25.0	47.5	11.5
	226	13.1	47.7	13.3	22.2	3.8
	313	23.7	13.8	12.2	8.1	42.3
**Nd2**	0	23.4	28.4	33.6	7.5	7.1
	40	20.8	6.3	26.1	19.2	27.6
	77	18.0	3.7	10.2	48.9	19.2
	214	17.6	39.6	20.0	10.4	12.4
	293	20.2	22.0	10.0	14.0	33.8

Table S13 Angles between the principal directions of g tensors calculated by ab initio and obtained by best fit of CTM curves, for the two crystallographically inequivalent lanthanides of **1** and **2**

		**1**	**2**
**Ln1**	g_x	29.4°	83.7°
	g_y	27.9°	83.8°
	g_z	17.1°	3.5°
**Ln2**	g_x	21.6°	49.4°
	g_y	20.9°	47.6°
	g_z	10.9°	13.9°

Ab Initio CASSCF/NEVPT2 Calculations with ORCA Program Package

We performed with ORCA 4.2 software CASSCF calculations followed by NEVPT2 second order perturbations. Active space and number of requested roots were the same ones used for the MOLCAS calculations. We employed SARC2-DKH-QZVP basis set for Ce, Nd and La;[1] DKH-def2-TZVP for Cl, O and N; DKH-def2-SVP for C and H;[2] SARC/J, Def2/J and def2/JK auxiliary basis sets[3–5] have been also employed within the RIJCOSX approximation.[6]

The CASSCF calculations by ORCA are directly comparable with Molcas result. The agreement among the two softwares is excellent for Nd. For Ce larger differences are found, even if not significant. Generally, the ground Kramers' g-tensor is found more axial in comparison with the MOLCAS results. However the tensor orientation inside the cell practically does not change and the energy level spacing is almost the same within few wavenumbers.

Once we showed that also ORCA provides reliable results for our systems, CASSCF+NEVPT2 simulations have been performed. The results showed that dynamical correlation included by second order perturbation theory did not improve significantly the agreement with experiment relatively to ground state anisotropy. The orientations of the ground Kramers'doublet g-tensor are not significantly affected even in these cases. Regarding the main g-values, our CASSCF/NEVPT2 calculations provided even more axial ground g-tensors, therefore they are not going in the direction of a more accurate reproduction of the experimental g-tensors. Indeed, in the paper we are claiming an overestimation of the axiality of the ground Kramers' doublet in both compounds by ab initio calculations, and such an overestimation is found even more pronounced in these last NEVPT2 results.

As already observed in literature,[7,8] dynamical perturbation significantly influences the energy of the higher excited states, in a larger amount starting from the second excited doublet both for Nd and Ce. However, such an energy increase should not improve the fit of magnetic data. Indeed EPR and cantilever magnetometry has been performed at very low temperatures, below 20 K, and as a consequence the shift at higher energies should not alter the simulation of the experimental results which can be, in first approximation, determined by the ground state anisotropy tensor.

Table S14. *g*-tensor main values and orientations of the main magnetic axes in the crystallographic frame for **Nd1** ground Kramers'doublets, computed with different softwares and level of theory

Software/Level of theory	***g*-tensor**		Orientations of main magnetic axes		
		Main Values	a	b'	c*
MOLCAS/CASSCF	g_x	0.586	-0.261453	-0.713044	0.650547
	g_y	1.857	-0.208554	-0.616337	-0.759364
	g_z	3.970	0.942416	-0.334212	0.012435
ORCA/CASSCF	g_x	0.539673	0.2891906	0.6632160	-0.6902994
	g_y	1.700259	-0.1563178	-0.6787148	-0.7175729
	g_z	4.060576	-0.9444223	0.3154214	-0.0926055
ORCA/CASSCF+NEVPT2	g_x	0.285988	0.2601786	0.6490345	-0.7148855
	g_y	1.660648	-0.1677258	-0.6987485	-0.6954269
	g_z	3.831434	-0.9508812	0.3008400	-0.0729395

Table S15. Energy levels (cm^{-1}) for **Nd1**, computed with different softwares and level of theory

MOLCAS/CASSCF	ORCA/CASSCF	ORCA/CASSCF+NEVPT2
0	0	0
37	37	43
85	89	108
226	218	274
313	310	378

Table S16. *g*-tensor main values and orientations of the main magnetic axes in the crystallographic frame for **Nd2** ground Kramers'doublets, computed with different softwares and level of theory

Software/Level of theory	*g*-tensor		Orientations of main magnetic axes		
		Main Values	a	b'	c*
MOLCAS/CASSCF	g_x	0.743	-0.126536	-0.647058	0.751868
	g_y	2.028	-0.111848	-0.743820	-0.658955
	g_z	3.925	0.985636	-0.167477	0.021748
ORCA/CASSCF	g_x	0.703787	0.1596927	0.5858598	-0.7945229
	g_y	1.952864	-0.0409277	-0.8002304	-0.5982945
	g_z	3.974211	-0.9863180	0.1280612	-0.1038130
ORCA/CASSCF+NEVPT2	g_x	0.550777	0.1698064	0.5865345	-0.7919236
	g_y	1.817701	0.0604515	-0.8082801	-0.5856867
	g_z	3.893465	-0.9836216	0.0515804	-0.1727080

Table S17. Energy levels (cm^{-1}) for **Nd2**, computed with different softwares and level of theory

MOLCAS/CASSCF	ORCA/CASSCF	ORCA/CASSCF+NEVPT2
0	0	0
40	40	46
77	81	99
214	207	260
293	290	353

Table S18. *g*-tensor main values and orientations of the main magnetic axes in the crystallographic frame for **Ce1** ground Kramers'doublets, computed with different softwares and level of theory

Software/Level of theory	***g*-tensor**		Orientations of main magnetic axes		
		Main Values	a	b'	c*
MOLCAS/CASSCF	g_x	0.527	-0.539549	-0.813079	-0.218609
	g_y	0.894	0.735706	-0.581551	0.347182
	g_z	3.065	-0.409418	0.026490	0.911962
ORCA/CASSCF	g_x	0.346313	-0.6201951	-0.7468280	-0.2400125
	g_y	0.662424	0.6678799	-0.6631945	0.3378008
	g_z	3.202757	-0.4114541	0.0492029	0.9101014
ORCA/CASSCF+NEVPT2	g_x	0.349618	-0.6159223	-0.7534746	-0.2300341
	g_y	0.626644	0.6738035	-0.6551309	0.3417488
	g_z	3.238339	-0.4082015	0.0554929	0.9112036

Table S19. Energy levels (cm^{-1}) for **Ce1**, computed with different softwares and level of theory

MOLCAS/CASSCF	ORCA/CASSCF	ORCA/CASSCF+NEVPT2
0	0	0
348	352	378
432	428	470

Table S20. g-tensor main values and orientations of the main magnetic axes in the crystallographic frame for **Ce2** ground Kramers'doublets, computed with different softwares and level of theory.

Software/Level of theory	***g*-tensor**		Orientations of main magnetic axes		
		Main Values	a	b'	c*
MOLCAS/CASSCF	g_x	0.272	-0.680434	-0.403074	-0.611997
	g_y	0.623	0.296825	-0.915159	0.272726
	g_z	3.066	-0.670003	0.003916	0.742348
ORCA/CASSCF	g_x	0.038175	-0.7212112	-0.3953523	-0.5688154
	g_y	0.419093	-0.2944017	0.9182261	-0.2649313
	g_z	3.155792	0.6270423	-0.0236112	-0.7786273
ORCA/CASSCF+NEVPT2	g_x	0.063518	-0.7138569	-0.4155138	-0.5636990
	g_y	0.407459	-0.3083812	0.9092205	-0.2796769
	g_z	3.171336	0.6287363	-0.0258151	-0.7771900

Table S21. Energy levels (cm^{-1}) for **Ce2**, computed with different softwares and level of theory

MOLCAS/CASSCF	ORCA/CASSCF	ORCA/CASSCF+NEVPT2
0	0	0
320	306	339
431	427	472

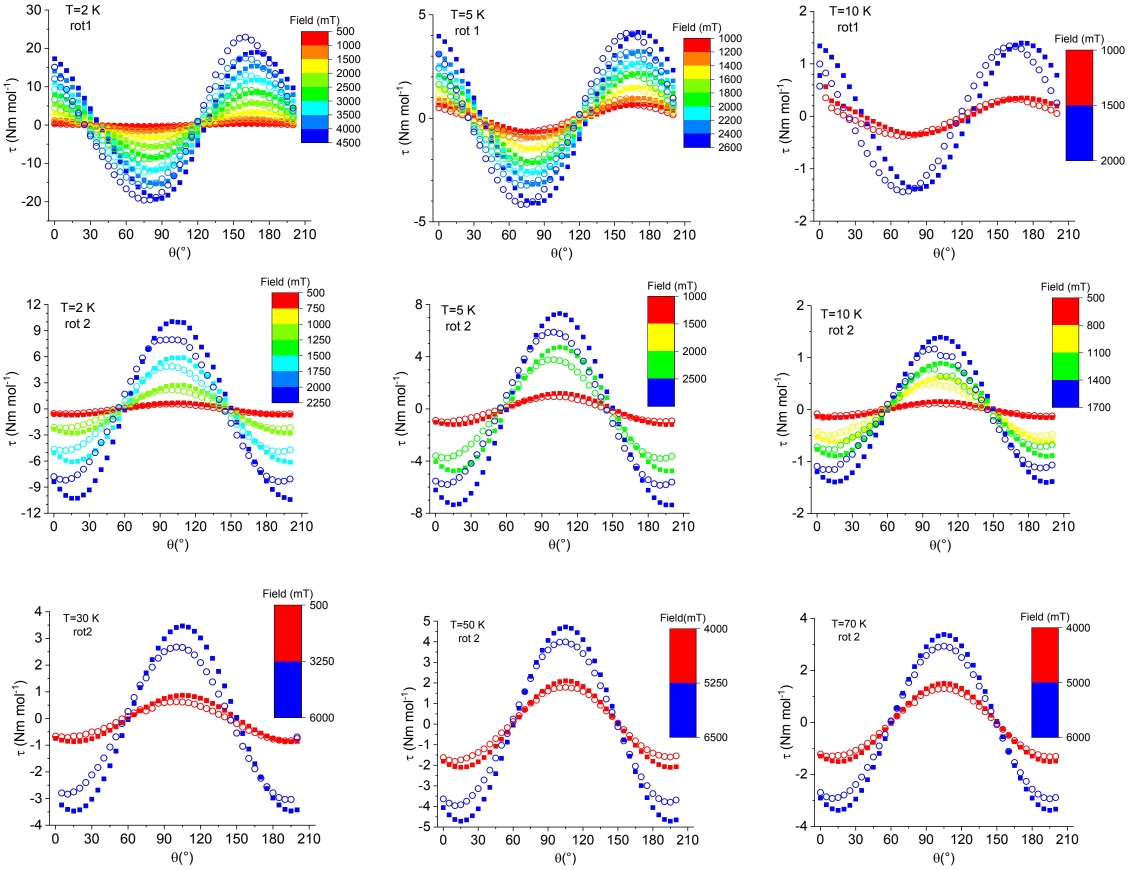

Figure S9 Comparison between ab initio calculated (empty circles) and experimentally measured magnetic torque for **1** at different fields and temperatures.

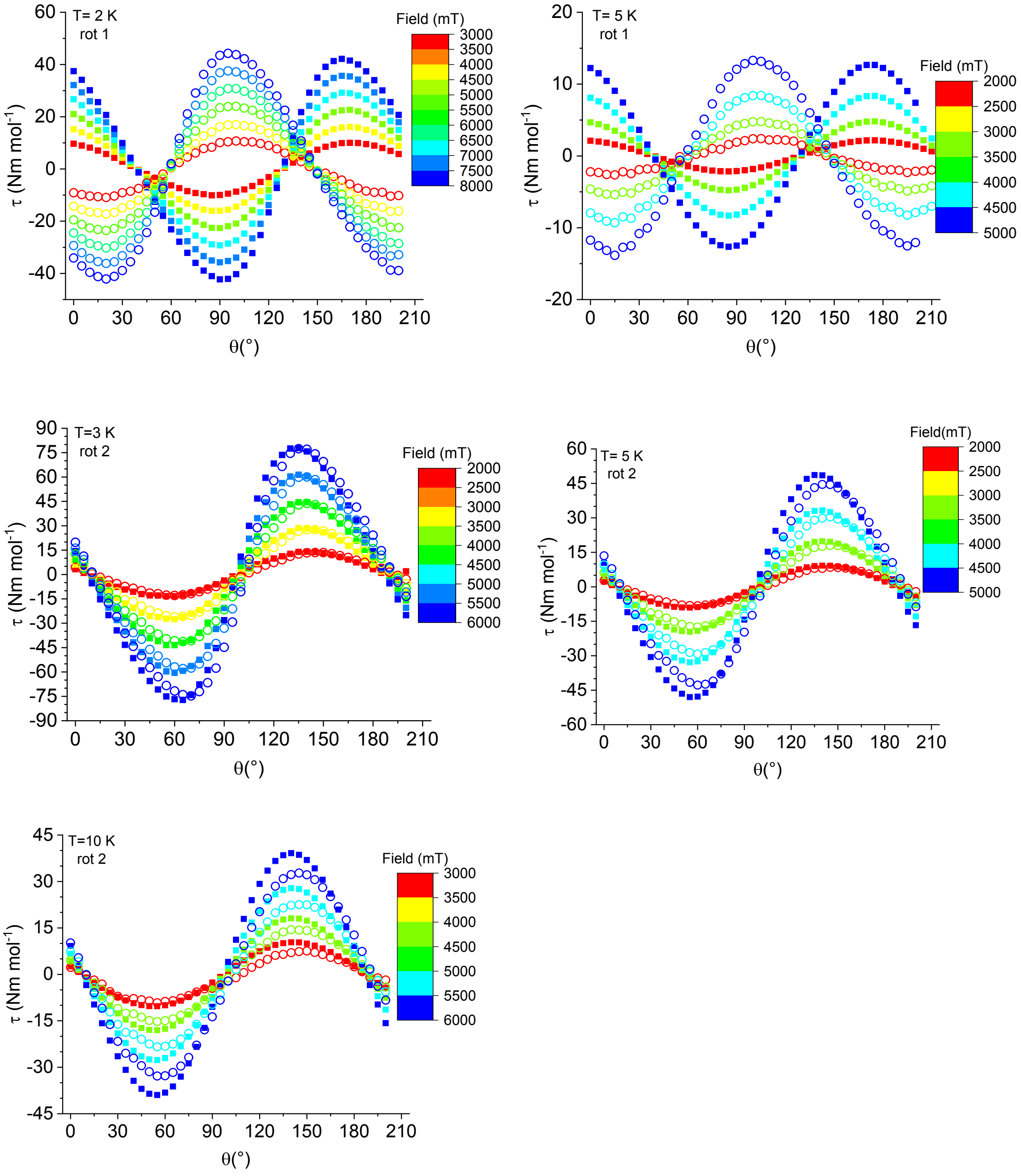

Figure S10 Comparison between ab initio calculated (empty circles) and experimentally measured magnetic torque for **2** at different fields and temperatures.

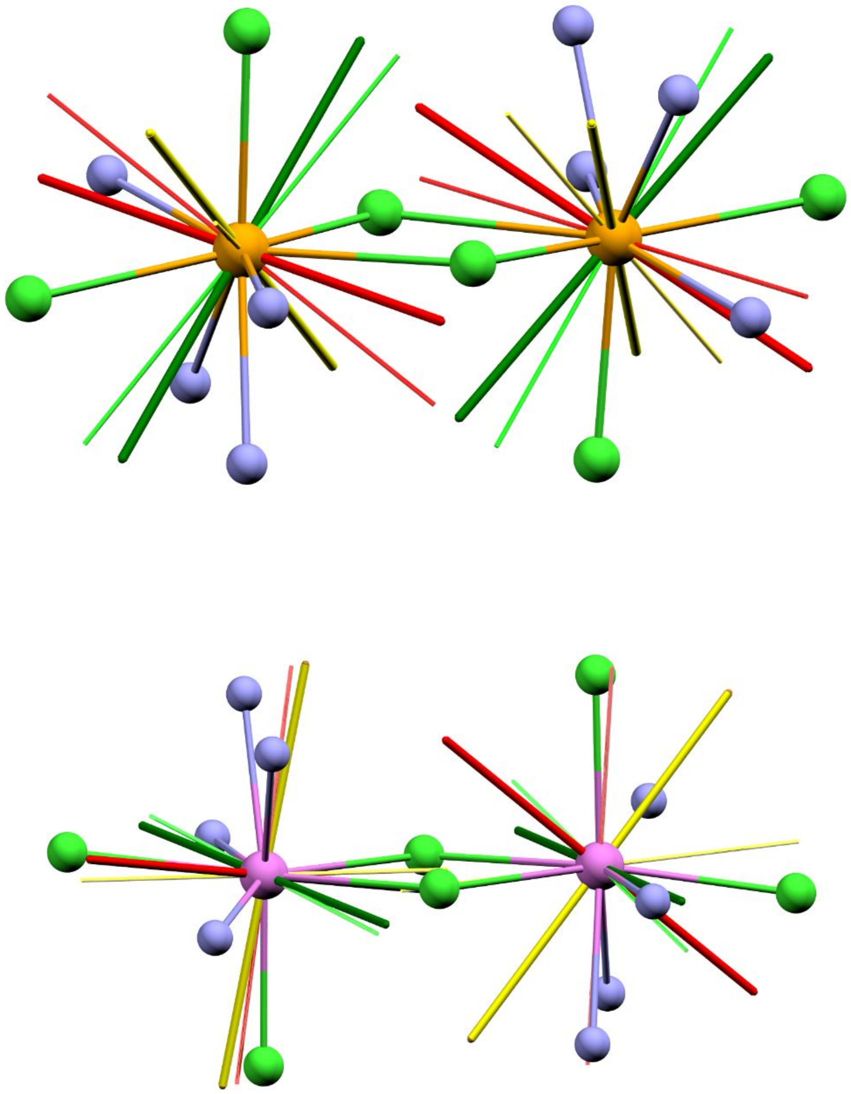

Figure S11 Comparison between the ab initio calculated (thin rods) principal directions of the g tensors of the ground doublets of the paramagnetic centers and those obtained by fitting the CTM data (thick rods) with two different centers (see text for details). Upper panel: **1**. Lower panel: **2**

AC susceptibility

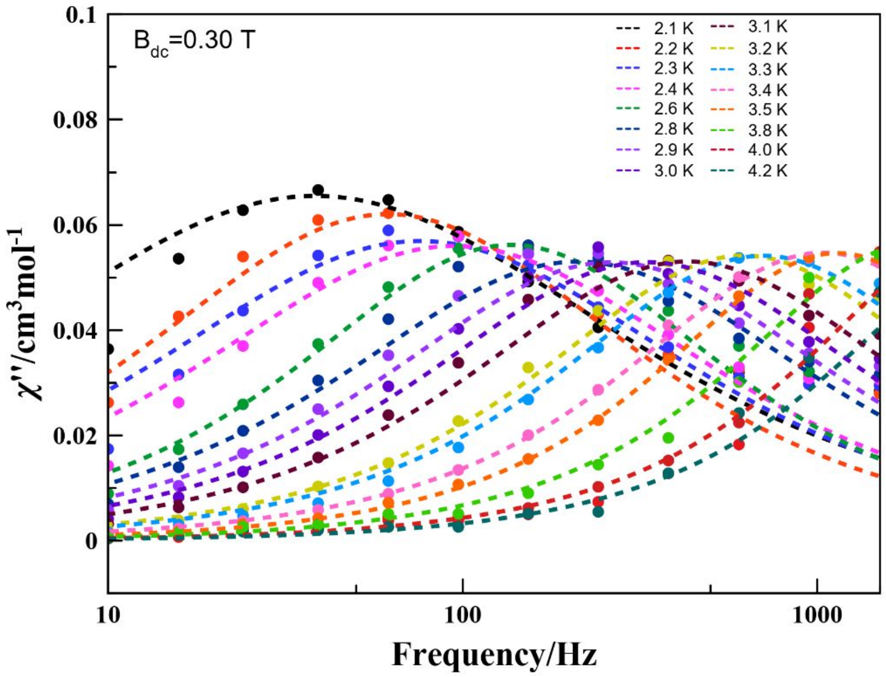

Figure S12. Frequency dependence of χ_M'' for **1** at different temperatures under a dc field of 0.30 T. Dashed lines are best fit using extended Debye model

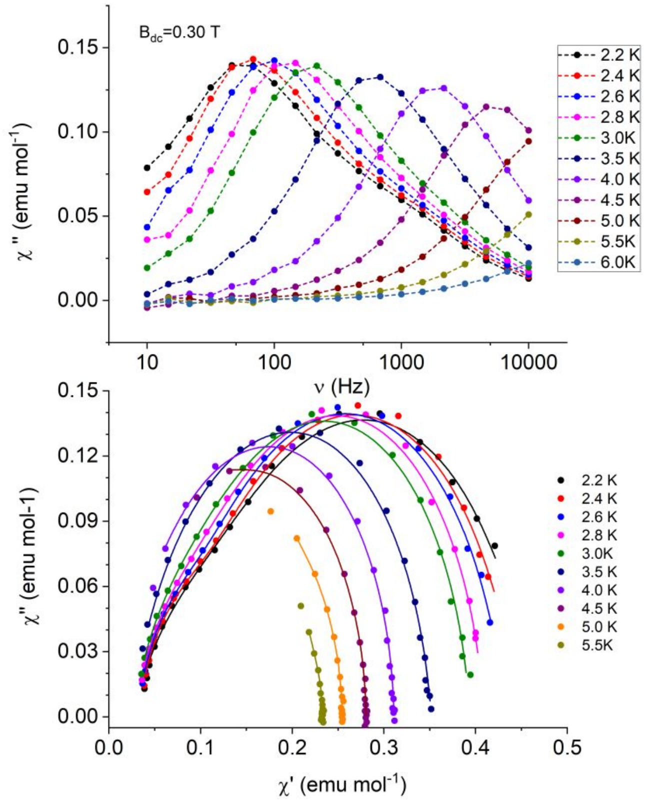

Figure S13. Upper: frequency dependence of χ_M'' for **2** at different temperatures under a dc field of 0.30 T. Dashed lines are guide to the eye. Lower: corresponding Cole-Cole plots and best fit obtained using extended Debye model.

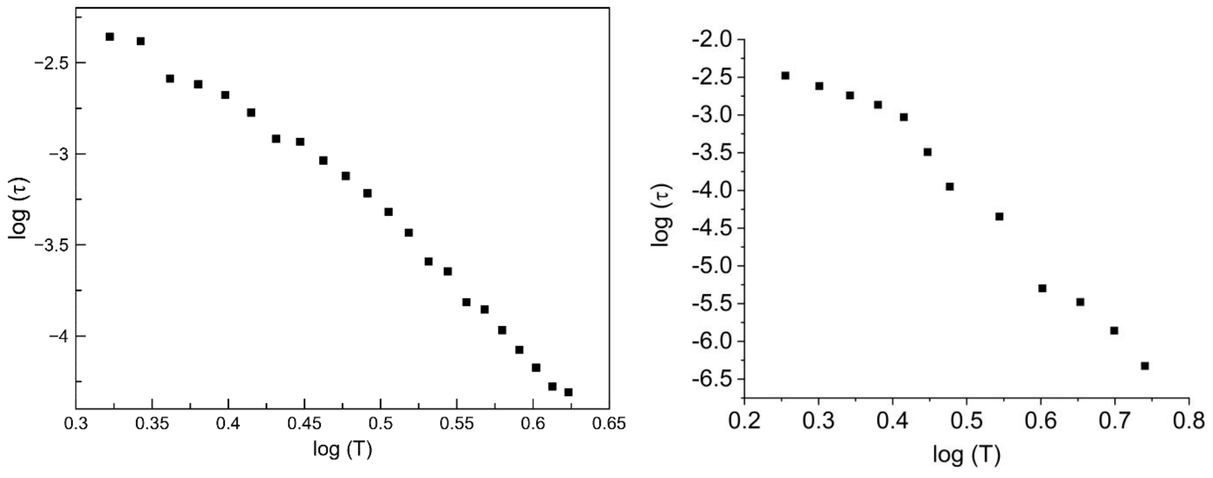

Figure S14. Temperature dependence of the relaxation time of 1(left) and **2** (right) plotted in log-log scale. The two different slopes at low and high temperature hints at dominant direct and Raman processes in the two temperature regions.

Computational details

Table S22 Basis sets and contractions employed for the CASSCF calculations.

**Atom**	**Label**	**Primitives**	**Contraction**
Ce, Nd	VTZP	[25s22p15d11f4g2h]	[8s7p5d3f2g1h]
La	VTZP	[25s22p15d11f4g2h]	[8s7p5d3f2g1h]
N	VTZP	[14s9p4d3f2g]	[4s3p2d1f]
Cl	VTZP	[14s9p4d3f2g]	[4s3p2d1f]
C	VDZ	[14s9p4d3f2g]	[3s2p]
H	VDZ	[8s4p3d1f]	[2s]

References

(1) Aravena, D.; Neese, F.; Pantazis, D. A. Improved Segmented All-Electron Relativistically Contracted Basis Sets for the Lanthanides. *J. Chem. Theory Comput.* **2016**, *12* (3), 1148–1156. https://doi.org/10.1021/acs.jctc.5b01048.

(2) Weigend, F.; Ahlrichs, R. Balanced Basis Sets of Split Valence, Triple Zeta Valence and Quadruple Zeta Valence Quality for H to Rn: Design and Assessment of Accuracy. *Phys. Chem. Chem. Phys.* **2005**, *7* (18), 3297–3305. https://doi.org/10.1039/b508541a.

(3) Weigend, F. Accurate Coulomb-Fitting Basis Sets for H to Rn. *Phys. Chem. Chem. Phys.* **2006**, *8* (9), 1057–1065. https://doi.org/10.1039/b515623h.

(4) Pantazis, D. A.; Neese, F. All-Electron Scalar Relativistic Basis Sets for the Lanthanides. *J. Chem. Theory Comput.* **2009**, *5* (9), 2229–2238. https://doi.org/10.1021/ct900090f.

(5) Weigend, F. Hartree–Fock Exchange Fitting Basis Sets for H to Rn. *J. Comput. Chem.* **2008**, *29* (2), 167–175. https://doi.org/10.1002/jcc.20702.

(6) Neese, F.; Wennmohs, F.; Hansen, A.; Becker, U. Efficient, Approximate and Parallel Hartree-Fock and Hybrid DFT Calculations. A "chain-of-Spheres" Algorithm for the Hartree-Fock Exchange. *Chem. Phys.* **2009**, *356* (1–3), 98–109. https://doi.org/10.1016/j.chemphys.2008.10.036.

(7) Hallmen, P. P.; Rauhut, G.; Stoll, H.; Mitrushchenkov, A. O.; Van Slageren, J. Crystal Field Splittings in Lanthanide Complexes: Inclusion of Correlation Effects beyond Second Order Perturbation Theory. *J. Chem. Theory Comput.* **2018**, *14* (8), 3998–4009. https://doi.org/10.1021/acs.jctc.8b00184.

(8) Ungur, L.; Chibotaru, L. F. Ab Initio Crystal Field for Lanthanides. *Chem. - A Eur. J.* **2017**, *23* (15), 3708–3718. https://doi.org/10.1002/chem.201605102.
